# Genes Interacting with Occupational Exposures to Low Molecular Weight Agents and Irritants on Adult-Onset Asthma in Three European Studies

**DOI:** 10.1289/EHP376

**Published:** 2016-08-09

**Authors:** Marta Rava, Ismail Ahmed, Manolis Kogevinas, Nicole Le Moual, Emmanuelle Bouzigon, Ivan Curjuric, Marie-Hélène Dizier, Orianne Dumas, Juan R. Gonzalez, Medea Imboden, Amar J. Mehta, Pascale Tubert-Bitter, Jan-Paul Zock, Deborah Jarvis, Nicole M. Probst-Hensch, Florence Demenais, Rachel Nadif

**Affiliations:** 1 Inserm, U1168, VIMA: Aging and Chronic Diseases, Epidemiological and Public Health Approaches, Villejuif, France; 2 Spanish National Cancer Research Centre (CNIO), Genetic and Molecular Epidemiology Group, Human Cancer Genetics Program, Madrid, Spain; 3 Inserm UMR 1181 [Biostatistics, Biomathematics, Pharmacoepidemiology and Infectious Diseases (B2PHI)], Villejuif, France; 4 Institut Pasteur, UMR 1181, B2PHI, Paris, France; 5 Univ Versailles St.-Quentin-en-Yvelines, UMR 1181, B2PHI, Montigny le Bretonneux, France; 6 ISGlobal, Centre for Research in Environmental Epidemiology (CREAL), Barcelona, Spain; 7 CIBER Epidemiología y Salud Pública, Madrid, Spain; 8 Univ Versailles St.-Quentin-en-Yvelines, UMR-S 1168, Montigny le Bretonneux, France; 9 Inserm, UMR-946, Genetic Variation and Human Diseases Unit, Paris, France; 10 Univ Paris Diderot, Sorbonne Paris Cité, Institut Universitaire d’Hématologie, Paris, France; 11 Department of Epidemiology and Public Health, Swiss Tropical and Public Health Institute, Basel, Switzerland; 12 University of Basel, Switzerland; 13 Department of Environmental Health, Harvard School of Public Health, Boston, Massachusetts, USA; 14 Universitat Pompeu Fabra (UPF), Barcelona, Spain; 15 Respiratory Epidemiology and Public Health, Imperial College, London, United Kingdom; 16 MRC-HPA (Medical Research Council and Health Protection Agency) Centre for Environment and Health, London, United Kingdom

## Abstract

**Background:**

The biological mechanisms by which cleaning products and disinfectants—an emerging risk factor—affect respiratory health remain incompletely evaluated. Studying genes by environment interactions (G × E) may help identify new genes related to adult-onset asthma.

**Objectives:**

We identified interactions between genetic polymorphisms of a large set of genes involved in the response to oxidative stress and occupational exposures to low molecular weight (LMW) agents or irritants on adult-onset asthma.

**Methods:**

Our data came from three large European cohorts: Epidemiological Family-based Study of the Genetics and Environment of Asthma (EGEA), Swiss Cohort Study on Air Pollution and Lung and Heart Disease in Adults (SAPALDIA), and European Community Respiratory Health Survey in Adults (ECRHS). A candidate pathway–based strategy identified 163 genes involved in the response to oxidative stress and potentially related to exposures to LMW agents/irritants. Occupational exposures were evaluated using an asthma job-exposure matrix and job-specific questionnaires for cleaners and healthcare workers. Logistic regression models were used to detect G × E interactions, adjusted for age, sex, and population ancestry, in 2,599 adults (mean age, 47 years; 60% women, 36% exposed, 18% asthmatics). p-Values were corrected for multiple comparisons.

**Results:**

Ever exposure to LMW agents/irritants was associated with current adult-onset asthma [OR = 1.28 (95% CI: 1.04, 1.58)]. Eight single nucleotide polymorphism (SNP) by exposure interactions at five loci were found at p < 0.005: PLA2G4A (rs932476, chromosome 1), near PLA2R1 (rs2667026, chromosome 2), near RELA (rs931127, rs7949980, chromosome 11), PRKD1 (rs1958980, rs11847351, rs1958987, chromosome 14), and PRKCA (rs6504453, chromosome 17). Results were consistent across the three studies and after accounting for smoking.

**Conclusions:**

Using a pathway-based selection process, we identified novel genes potentially involved in adult asthma by interaction with occupational exposure. These genes play a role in the NF-κB pathway, which is involved in inflammation.

**Citation:**

Rava M, Ahmed I, Kogevinas M, Le Moual N, Bouzigon E, Curjuric I, Dizier MH, Dumas O, Gonzalez JR, Imboden M, Mehta AJ, Tubert-Bitter P, Zock JP, Jarvis D, Probst-Hensch NM, Demenais F, Nadif R. 2017. Genes interacting with occupational exposures to low molecular weight agents and irritants on adult-onset asthma in three European studies. Environ Health Perspect 125:207–214; http://dx.doi.org/10.1289/EHP376

## Introduction

Recent reviews regarding the role of environmental risk factors in adult-onset asthma showed that occupational exposures are important causes of asthma in adults (Le Moual et al. 2013; Beasley et al. 2015). Approximately 15% of adult asthma is likely to be attributable to occupational exposures (Torén and Blanc 2009), and occupational asthma is known to be a good model for studying the pathophysiology of asthma in general (Malo et al. 2015). Exposure to cleaning agents is an emerging risk factor for adult-onset asthma. Evidence of adverse effects of cleaning products and disinfectants in asthma mostly comes from studies on occupational risk factors (Siracusa et al. 2013), but a deleterious role has also been observed for domestic cleaning exposure (Quinn et al. 2015; Le Moual et al. 2013; Dumas et al. 2013). Some of the numerous agents contained in cleaning products and disinfectants are chemical sensitizers, but most are hypothesized to act as respiratory irritants (Siracusa et al. 2013). The biological mechanisms by which cleaning products and disinfectants affect respiratory health remain incompletely evaluated (Tarlo and Lemiere 2014; Le Moual et al. 2013; Tarlo 2014). However, inhalation of low molecular weight (LMW) agents and irritants is likely to induce the release of reactive oxygen species through the epithelium, and oxidative stress is known to be a potential mechanism of epithelium injury (Mittal et al. 2014). Furthermore, there is strong evidence that an imbalance between the reducing and oxidizing systems favoring the oxidative state is present in asthma. Reactive oxygen and nitrogen species from endogenous and exogenous sources play major roles in airway inflammation, and oxidative stress is an important pathophysiological component of asthma (Chung and Marwick 2010; Aldakheel et al. 2016). Thus, to better understand the mechanism by which LMW chemical sensitizers and irritants are related to asthma, it may be particularly relevant to focus on the oxidative pathway (Tarlo and Lemiere 2014; Tarlo 2014).

Asthma is a heterogeneous disease, and it is now well established that it is caused by a complex interplay of environmental and genetic factors (Kauffmann and Demenais 2012). Considerable efforts have been made to characterize the genetic determinants of asthma (Holloway et al. 2010); however, the identified genetic factors explain only a small part of the genetic component of asthma. One reason for this lack of understanding is that many genetic factors are likely to be involved in the development, activity, and severity of asthma, and that these factors act primarily through complex mechanisms that involve interactions with environmental factors (G × E) and with other genes (G × G), notably through pathways and networks. Furthermore, the effects of such genetic factors may be missed if genes are considered individually, regardless of the biological functions they share with other genes or the pathways in which they are involved (Liu et al. 2012). Candidate G × E interaction studies conducted on genes involved in the response to oxidative/nitrosative stress and their interaction with environmental exposures in asthma focused more on children than on adults and mostly focused on outdoor air pollution and smoking (Romieu et al. 2010; Minelli et al. 2011). Furthermore, these studies have explored a limited number of genes (Kauffmann and Demenais 2012; Kogevinas 2014; Rava et al. 2015). To increase the number of genes to be investigated, we recently proposed a candidate pathway–based strategy to select an enriched gene set for G × E interaction studies (Rava et al. 2013). This gene selection process integrates information on the biological processes shared by genes, the canonical pathways to which genes belong, and the biological knowledge related to the environmental exposure under study. This approach represents a powerful alternative strategy between genome-wide and candidate approaches to detect relevant associations of environmental exposures with biological markers as well as G × E interactions.

In the present paper, we hypothesized that genes involved in the response to oxidative stress modify the associations of exposure to LMW agents and irritants with current asthma. We first applied the candidate pathway–based strategy to select oxidative stress–related genes that may interact with occupational exposures to LMW agents and irritants in current adult-onset asthma. We then tested for interactive effects of single nucleotide polymorphisms (SNPs) of these genes and LMW agents and irritants on current adult-onset asthma in 2,599 participants from the French Epidemiological family-based study of the Genetics and Environment of Asthma (EGEA), the Swiss Cohort Study on Air Pollution and Lung and Heart Disease in Adults (SAPALDIA), and the European Community Respiratory Health Survey (ECRHS).

## Methods

### Study Population

Our data came from three multicenter epidemiological European studies: EGEA (Kauffmann et al. 1997; Kennedy et al. 2000) (see Figure S1A) and two population-based studies, SAPALDIA (Downs et al. 2007; Mehta et al. 2012; Ackermann-Liebrich et al. 2005) (see Figure S1B) and ECRHS (ECRHS 2002; Kogevinas et al. 2007) (see Figure S1C). All three cohorts used comparable study designs and highly comparable questionnaires. Participants included in the analyses were derived from the entire study population for EGEA and from the nested case–control samples within the ECRHS (Smit et al. 2014) and SAPALDIA cohorts (Curjuric et al. 2012). Participants had genome-wide SNP data; occupational history regarding LMW agents and irritants, particularly cleaning/disinfecting products; and data on adult-onset asthma and relevant covariates such as age, sex, and smoking status.

Ethical approval was obtained for each study from the appropriate institutional ethics committees, and written informed consent was obtained from each participant. Detailed cohort descriptions are given in the Supplemental Material, “Description of the three multicentre epidemiological European studies.”

### Current Adult-Onset Asthma

In all cohorts, current asthma was defined as ever diagnosis of asthma (Moffatt et al. 2010; Smit et al. 2014) and presence of respiratory symptoms (wheeze; nocturnal chest tightness; attacks of breathlessness after activity, at rest, or at night; asthma attacks) or using asthma medications in the last 12 months. Participants without asthma were those without asthma at baseline and at follow-up. Participants with ever asthma but without symptoms or treatment in the last 12 months were excluded. Because we were interested in participants who may have developed asthma because of occupational exposure, we restricted the current adult-onset asthma definition to asthmatics with an age of onset ≥ 16 years old.

### Occupational Exposures to LMW Agents and Irritants

In all cohorts, occupational history was recorded by interview, and job codes were linked to an asthma-specific job-exposure matrix (JEM) evaluating exposure to 22 agents and including a local expert reevaluation step (Kennedy et al. 2000). Healthcare workers and cleaners were further asked to answer a job-specific questionnaire regarding exposure to cleaning/disinfecting products.

In the present study, we considered only exposures to substances hypothesized to cause irritant-asthma or to cause asthma through mechanisms induced by LMW agents. Exposure to LMW agents was evaluated by the JEM and included not only products typically classified as LMW agents (e.g., highly reactive chemicals, metals) but also mixed environments with potential exposure to high molecular weight (HMW) and LMW agents (e.g., agriculture, textiles). Exposure to irritants was evaluated using *a*) the JEM, for high-peak irritant exposure, and *b*) self-reported exposure to cleaning/disinfecting products, with a focus on those with a greater likelihood of being respiratory irritants (see Table S1 for more details). Participants who had ever been exposed to any of the LMW agents, mixed environments, irritants, or any specific cleaning/disinfecting products were classified as “exposed.” Unexposed participants were those who were never exposed to any of the 22 agents of the asthma JEM (including HMW agents) or to other potential risk agents for respiratory health (vapors, general dusts, gases, and fumes) evaluated by another JEM (ALOHA JEM) (Matheson et al. 2005; de Jong et al. 2015). All three cohorts used the same definitions.

### Genotyping

The three cohorts (EGEA, SAPALDIA, and ECRHS) were part of the European Gabriel consortium (http://cordis.europa.eu/project/rcn/84712_en.html) for asthma genetics (Moffatt et al. 2010) and constitute the ESE consortium. Participants were genotyped using the Illumina 610 Quad array (Illumina, San Diego, CA) at the Centre National de Génotypage (CNG; Evry, France). Stringent quality criteria, as detailed by Imboden et al. (2012), were used to select both individuals and SNPs for analysis. The quality control (QC) criteria were call rate ≥ 97%, minor allele frequency ≥ 5%, and Hardy–Weinberg (HW) *p*-value > 10^–4^.

Gene coverage, which indicates the fraction of common HapMap markers successfully tagged by the set of selected SNPs, was obtained using Haploview 4.2 (Barrett et al. 2005). We specified that all HapMap markers being captured by the set of tags should be correlated at *r* ^2^ ≥ 0.8 with at least one marker in the set.

### Gene Selection Through a Candidate Pathway–Based Strategy

For this study, a large set of genes was selected according to a previously published candidate pathway–based strategy (Rava et al. 2013). Briefly, the selection process followed three steps.

***Step 1: Gene selection.*** We used the Gene Ontology (GO) database [Gene Ontology Consortium (Ashburner et al. 2000; http://amigo2.berkeleybop.org/amigo, version 1.8] to select genes involved in the response to oxidative stress (GO:0006979). This list was further enlarged by literature reviews of asthma-related genome-wide association studies and biological studies on response to oxidative stress related to environmental exposures of interest.

***Step 2: Pathway enrichment.*** Using Ingenuity Pathway Analysis (IPA; http://www.ingenuity.com/), we identified the canonical pathways that contained ≥ 5 genes out of the set of the genes selected in Step 1 and that were significantly enriched in these genes (*p* < 0.05).

***Step 3: Environment integration.*** We selected the subset of pathways identified at Step 2 that contained genes selected at Step 1 expected to be involved in the response to oxidative stress potentially caused by occupational exposure to LMW agents or irritants. This strategy has been fully detailed by Rava et al. (2013).

For each of the genes belonging to the selected pathways, we examined all SNPs passing the QC process and lying from 20 kb upstream to 20 kb downstream of the gene [UCSC genome browser hg18 assembly, build 37.1 (Kent et al. 2002; https://genome.ucsc.edu/)]).

### Statistical Analysis Strategy

The three ESE cohorts were pooled to increase statistical power as before (Smit et al. 2012, 2014); this strategy also allowed us to assess the consistency of the results across cohorts. SNP–occupational exposure interactions were investigated using a logistic regression model that included the SNP effect assumed to be additive, a binary exposure (E) variable (1 = exposed, 0 = unexposed), and a multiplicative term for SNP × E interaction. All models were adjusted for age, sex, and the four first principal components (PCs) to account for population stratification as was done previously (Smit et al. 2014). No additional adjustments were made for study because PCs capture any possible variability caused by geographical location. Smoking status was further included as a potential confounder.

The test of SNP × E interaction was based on a Wald test. To account for multiple testing, the Benjamini–Hochberg (1995) procedure was implemented. For interactions belonging to the top 1% of *p*-value distributions, the consistency of interaction effect estimates across studies was assessed using Cochran’s Q test, and the extent of heterogeneity was measured using *I* ^2^, which ranges from 0% to 100%. The *I* ^2^ statistic describes the percentage of variation across studies that is due to heterogeneity rather than to chance (Higgins and Thompson 2002; Higgins et al. 2003); *I* ^2^ = 100% × (Q-degree of freedom)/Q. *I* ^2^ values of 0–24% suggest little heterogeneity; values of 25–49% reflect moderate heterogeneity; values of 50–74% reflect large heterogeneity; and *I* ^2^values > 75% reflect very large heterogeneity (Viechtbauer and Cheung 2010). Because smoking may also induce oxidative stress, a sensitivity analysis excluding current smokers was performed. The robustness of the results to the family dependency existing in the EGEA study was investigated using generalized estimating equations (GEE) with an exchangeable working correlation matrix to take into account potential clustering within families.

For each of the genes belonging to the selected pathways, interactions with occupational exposure for current adult-onset asthma were also investigated at the gene level using the versatile gene-based test (VEGAS; Liu et al. 2010). This gene-based statistic sums up the χ^2^ test statistics of SNP × E interactions (square of the Wald test statistics) for all SNPs of a gene. The correlation (*r* ^2^) between these statistics is taken into account by computing an empirical *p*-value through Monte Carlo simulations using the linkage disequilibrium pattern of HapMap Utah residents with ancestry from northern and western Europe (CEU) reference sample; this empirical *p*-value is estimated by the proportion of simulated test statistics that exceeds the observed gene-based test statistic. The empirical *p*-values were then adjusted for multiple testing using the method of Benjamini and Hochberg (1995).

### Expression Quantitative Trait Loci Analysis, Functional Annotation and Chemical–Gene/Protein Interactions

We investigated whether the SNPs (or their proxies, *r* ^2^ ≥ 0.8) found to interact with occupational exposures to LMW agents or irritants were cis-expression quantitative trait loci (cis-eQTLs). We used the eQTL browser (http://www.gtexportal.org/home/), which includes eQTL data from many tissues from the Genotype–Tissue Expression project (GTEx) (Gibson 2015). Furthermore, functional annotations of these SNPs (or proxies) were made using the HaploReg tool (http://www.broadin​stitute.org/mammals/haploreg/haploreg.php). HaploReg annotates SNPs in terms of predicted ROADMAP and ENCyclopedia Of DNA Elements (ENCODE), chromatin states (promoter and enhancer histone modification signals), DNase I hypersensitivity sites, and transcription factor (TF) and protein binding sites.

Furthermore, curated (chemical–gene interactions/chemical–disease/gene–disease) data were retrieved from the Comparative Toxicogenomics Database (CTD; http://ctdbase.org/; Davis et al. 2015; MDI Biological Laboratory, Salisbury Cove, Maine, and North Carolina State University, Raleigh, North Carolina). CTD is a robust, publicly available database that aims to advance understanding about how environmental exposures affect human health. It provides manually curated information about chemical–gene/protein interactions and chemical–disease and gene–disease relationships.

## Results

### Data Description

The study population included 2,599 participants with a mean age of 46.7 years and 60% women (Table 1). ECRHS participants were younger than SAPALDIA and EGEA participants, and the proportion of women was lower in SAPALDIA. Almost half of the participants were never smokers. The proportion of current smokers varied from 18.6% (EGEA) to 31.4% (ECRHS), and 463 had current adult-onset asthma. Among the 927 exposed participants, 25.4% were exposed to LMW agents only, 4.4% were exposed to irritants only, 23.7% were health care workers or cleaners (exposure to cleaning products), 12.6% were exposed to mixed environment only, and 33.9% had combined exposures (i.e., two or more of the aforementioned exposures).

**Table 1:** Characteristics of adult participants in the three studies.

Characteristic	All (*n*= 2,599)	ECRHS (*n *= 1,336)	SAPALDIA (*n *= 574)	EGEA (*n *= 689)
Age, year, mean (SD)	46.7 (11.3)	43.1 (7.1)	53.4 (10.9)	48.0 (14.9)
Sex, women, *n* (%)	1,563 (60.1)	822 (61.5)	311 (54.2)	430 (62.4)
Smoking habits, *n* (%)
Never smokers	1,167 (44.9)	569 (42.6)	248 (43.2)	350 (50.8)
Former smokers	735 (28.3)	337 (25.2)	191 (33.3)	207 (30.0)
Current smokers	682 (26.2)	419 (31.4)	135 (23.5)	128 (18.6)
Missing	15 (0.6)	11 (0.8)	0 (0.0)	4 (0.6)
Occupational exposure, *n* (%)^*a*^	927 (35.7)	440 (32.9)	175 (30.5)	312 (45.3)
Current adult-onset asthma, *n* (%)	463 (17.8)	234 (17.5)	107 (18.6)	122 (17.7)
Notes: ECHRS, European Community Respiratory Health Survey; EGEA, Epidemiological family-based study of the Genetics and Environment of Asthma; SAPALDIA, Swiss Cohort Study on Air Pollution and Lung and Heart Disease in Adults. ^***a***^Percent ever exposed to low molecular weight agents or to mixed environments or to high-peak irritants, or to specific cleaning products or disinfectants in the population selected for the analyses, that is to say, after excluding adults with occupational exposures to other potentially asthmagenic agents (high molecular weight agents).				

A positive and significant association was found between lifetime occupational exposure to LMW agents or irritants and current adult-onset asthma: age- and sex-adjusted pooled Odds Ratio (ORa) = 1.28; [95% confidence interval (CI): 1.04, 1.58]. Across the three cohorts, the associations between exposure and asthma were as follows: age- and sex-adjusted ORa = 1.09 (95% CI: 0.72, 1.65; *n* = 122/689, cases/all) in EGEA, 0.89 (95% CI: 0.56, 1.42; *n* = 107/574) in SAPALDIA, and 1.55 (95% CI: 1.15, 2.08; *n* = 234/1,336) in ECRHS.

### Genes Selected with the Candidate Pathway–Based Strategy

***Step 1: Gene selection.*** A total of 387 genes were selected through GO and further enriched by literature reviews and biological studies to obtain a list of 411 genes.

***Step 2: Pathway enrichment.*** We identified 277 pathways that contained ≥ 5 genes out of the 411 genes selected at step 1 and were enriched in these genes (*p* < 0.05).

***Step 3: Environment integration.*** Seventeen of the 277 pathways were further selected because they included genes involved in responding to oxidative stress and were potentially related to exposures to LMW agents or irritants. These pathways had pathway enrichment *p*-values ranging from 0.03 to 1.58 × 10^–31^ (see Excel File Table S1) and included 5–47 genes (15–20 genes on average); > 50% of the genes were involved in more than one pathway. The final analyzed set included a total of 163 unique genes (see Excel File Table S2) and 3,297 SNPs.

### Analysis of SNPs × Occupational Exposure Interactions

At the SNP level, none of the interactions with LMW/irritants on current adult-onset asthma reached the level of significance after correction for multiple testing (*p* = 0.05/3,297 = 1.5 × 10^–5^). However, we selected 14 interactions belonging to the top 1% of the *p*-value distribution ranked from lowest (top) to highest (bottom) (see Table S2). Among these 14 interactions, 8 interactions at 5 loci showed little heterogeneity (*I* ^2^ < 24%) between the three studies (Table 2; see also Table S3): rs932476 in *PLA2G4A* [phospholipase A2, group IVA (cytosolic, calcium-dependent) gene, chromosome 11, *p* = 0.005]; rs2667026 near *PLA2R1* (phospholipase A2 receptor 1, chromosome 2, *p* = 0.005); rs931127 and rs7949980 near *RELA* (v-rel avian reticuloendotheliosis viral oncogene homolog A gene; chromosome 11; *p* = 0.001 and *p* = 0.003, respectively), rs1958980, rs11847351, and rs1958987 in *PRKD1* (protein kinase D1, chromosome 14, *p*-values ranging from 0.004 to 0.005); and rs6504453 in *PRKCA* (protein kinase C alpha, chromosome 17, *p* = 0.003). The two SNPs near *RELA* were in moderate linkage disequilibrium (LD; *r* ^2^ = 0.65), whereas the three SNPs in *PRKD1* were in strong LD (*r* ^2^ > 0.8; see Figure S2A–E). Further, rs932476 in *PLA2G4A* and rs931127 in *RELA* were also marginally associated with asthma (*p* = 0.0036 and *p* = 0.035, respectively, Table 2). Similar interactive and marginal estimates were obtained by taking into account family dependency (see Table S4) or by adjusting for study/center (data not shown). Excluding current smokers from the analysis showed consistent results except for *PLA2G4A* (see Table S4). Finally, adjusting for smoking gave similar estimates (see Table S4).

**Table 2:** Interactive effects of single nucleotide polymorphisms with occupational exposure to low molecular weight agents or irritants on current adult-onset asthma.

Chr	Gene	SNP	Reference/effect allele	EAF^*a*^	Cases/controls *n*/*n*	Marginal effect		Interaction–CC	
						OR	*p*-Value	OR	*p*-Value
1	*PLA2G4A*	rs932476	A/G	0.35	463/2,136	1.25	0.0036	0.64	0.0050
2	*PLA2R1*	rs2667026	A/G	0.83	463/2,136	0.89	0.2354	1.77	0.0050
11	*RELA*^*b*^	rs931127	A/G	0.43	462/2,135	1.17	0.0350	1.61	0.0014
11	*RELA*	rs7949980	C/T	0.51	463/2,133	1.07	0.3421	1.56	0.0030
14	*PRKD1*^*b*^	rs1958980	A/G	0.67	463/2,136	1.08	0.3344	0.64	0.0042
14	*PRKD1*	rs11847351	A/G	0.67	463/2,133	1.08	0.3429	0.64	0.0043
14	*PRKD1*	rs1958987	C/T	0.68	459/2,127	1.07	0.3609	0.64	0.0050
17	*PRKCA*	rs6504453	C/T	0.35	462/2,134	1.04	0.6086	0.63	0.0032
Notes: CC, case–control; Chr, chromosome; EAF, effect allele frequency; OR, odds ratio; SNP, single nucleotide polymorphism. ^***a***^Calculated in controls. ^***b***^The two SNPs near *RELA* are in moderate linkage disequilibrium (LD) with *r*^2^ = 0.65, whereas the three SNPs in* PRKD1* are in strong LD (*r*^2^ > 0.8).									

**Figure 1:**
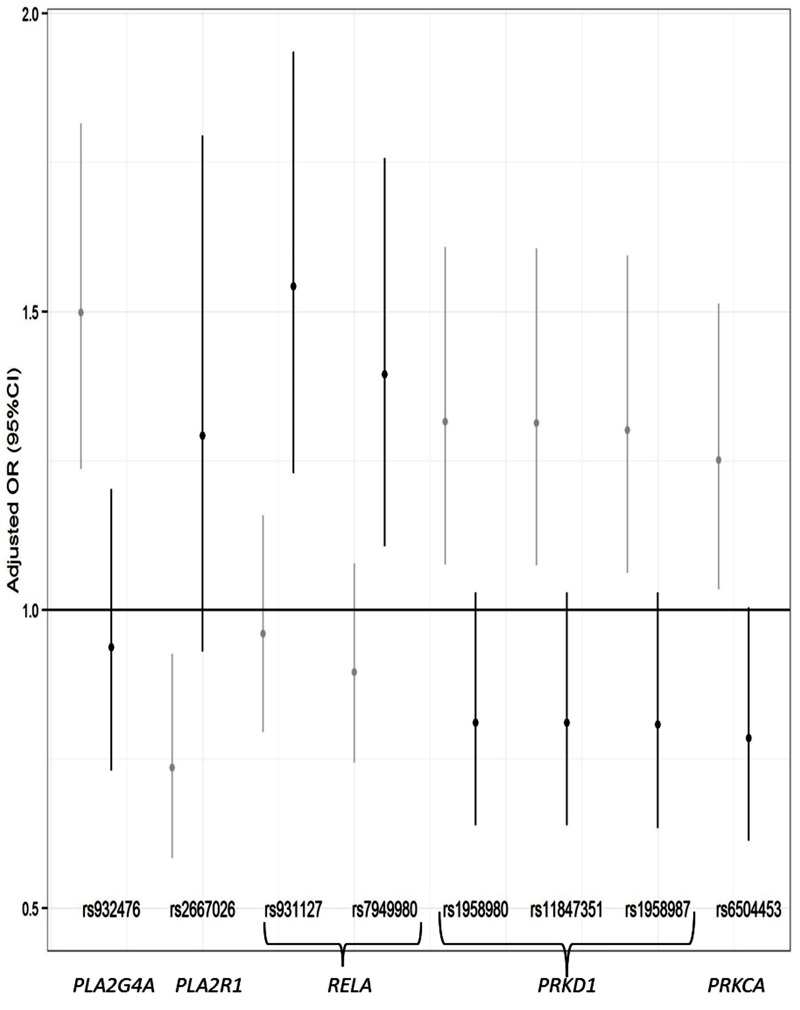
Associations between SNPs that showed an interactive effect with occupational exposure to low molecular weight agents or irritants on current adult-onset asthma in unexposed (gray) and exposed (black) participants.

Associations between SNPs and current adult-onset asthma in unexposed and exposed participants are reported in Figure 1. “Flip-flop” interactions were observed. Near *RELA*, the risk of current adult-onset asthma was increased in G carriers of rs931127 and in T carriers of rs7949980 among exposed participants (OR = 1.54, *p* = 2 × 10^–4^ and OR = 1.40, *p* = 0.005, respectively), whereas inverse but non-significant effects were observed among unexposed participants. The risk was also increased—although not statistically significant—among exposed participants for G carriers of rs2667042 near *PLA2R1*, whereas inverse and significant effects (OR = 0.74, *p* = 0.009) were observed among unexposed participants. On the contrary, the risk of current adult-onset asthma was decreased, but not significantly so, among exposed participants for G carriers of rs932476 in *PLA2G4A*; for G carriers of rs1958980, G carriers of rs11847351, or T carriers of rs1958987 in *PRKD1*; and for T carriers of rs6504453 in *PRKCA*; the risk was decreased significantly for T carriers of rs6504453 in *PRKCA* (OR = 0.79, *p* = 0.05), whereas inverse and significant effects were observed among unexposed participants (OR = 1.25 to 1.50, *p* = 0.01 to 3 × 10^–4^).

*PRKD1* and *PRKCA* are involved together in the NRF2-mediated oxidative stress response pathway; in association with *RELA* in three other pathways: xenobiotic metabolism signaling, production of nitric oxide and reactive oxygen species in macrophages, and *N*-formyl-methionine-leucyl-phenylalanine (fMLP) signaling in neutrophils; and in association with *PLA2G4A* in CCR3 signaling in eosinophils (see Excel File Table S3). Furthermore, *RELA* and *PRKCA* are involved together in apoptosis signaling, and *RELA*, *PLA2G4A,* and *PLA2R1* are involved together in the antioxidant action of vitamin C pathway.

Gene coverage for the SNPs in *PLA2G4A*, *PLA2R1*, *PRKD1*, and *PRKCA* was quite high: 55% (*r* ^2^ = 0.98), 76% (*r* ^2^ = 0.97), 74% (*r* ^2^ = 0.96) and 68% (*r* ^2^ = 0.96), respectively. Low coverage was observed for *RELA* (< 10%).

### Analysis of Gene × Occupational Exposure Interactions

At the gene level, *RELA* and *PRKD1* were among the top gene interactions with occupational exposures to LMW/irritants that were detected by the gene-based test among all 163 studied genes (*p*-value = 0.009 and *p* = 0.04, respectively; see Table S5), but none reached significance after correction for multiple testing.

### eQTL, Functional Annotations and Chemical–Gene/Protein Interactions

Using the eQTL browser GTEx, we found that the T allele at rs6504453 in *PRKCA* was associated with increased gene expression in lung tissue (see Figure S3, *p* = 0.017). No eQTL was found among the SNPs (or proxies) interacting with exposures at *PLA2G4A*, *PLA2R1*, *RELA* and *PRKD1* loci.

Using the functional annotation tool HaploReg v3, we found that the SNPs rs932476 in *PLA2G4A*, rs2667026 near *PLA2R1*, rs931127 and rs7949980 near *RELA*, and rs1958980, rs11847351, and rs1958987 in *PRKD1* mapped to marks in active regulatory elements, notably in B cells, small airway epithelial cells, and lymphoblastoid cell lines. These marks included enhancer histone marks, DNase hypersensitivity sites, and binding protein sites for NF-κB, histone deacetylase 2 (HDAC2), and nuclear factor erythroid 2-related factor 2 (Nrf2) (see Excel File Table S4).

Further, from the CTD (http://ctdbase.org/; Davis et al. 2015; MDI Biological Laboratory, Salisbury Cove, Maine, and North Carolina State University, Raleigh, North Carolina), we found that chlorine, formaldehyde, and hydrogen peroxide have been reported to modify the localization of *PRKCA* protein, the expression of *PLA2R1* and *PLA2G4A* mRNA, and the expression and activity of RELA protein (see Excel File Table S5). We also found that exposures known to contain compounds with irritant properties (air pollutants and vehicle emissions) modified the expression of *PRKD1* mRNA and the methylation of *PLA2R1* (see Excel File Table S5).

## Discussion

This study identified interactions between genetic variants near or within five genes, *PLA2G4A*, *PLA2R1*, *RELA*, *PRKD1* and *PRKCA,* and occupational exposures to LMW agents or irritants for current adult-onset asthma. The evidence for these interactions lies in the results obtained from the pooled data of three large European epidemiological studies and the consistency of the results across these studies. Functional annotations of the interacting SNPs at these loci and functional supports specific for the G × E interactions detected suggest that a few of these SNPs might be involved in regulatory mechanisms.

Until now, a limited number of genes were explored in G × E interaction studies conducted with candidate gene approaches. The most commonly studied genes were those coding for the enzymes NAD(P)H dehydrogenase [quinine] 1 (*NQO1*), the glutathione *S*-transferases (*GST*s), heme oxygenase 1 (*HMOX-1*), catalase (*CAT*) and superoxide dismutase (*SOD*) (Minelli et al. 2011). Our study relied on an original strategy to select and enlarge the list of candidate genes. Because it is supported by biological knowledge, this approach allows a good tradeoff between genome-wide interaction studies (GEWIS) and candidate gene approaches. It is interesting to note that our set of 163 genes included the few genes mentioned previously and studied in interaction with other exposures related to oxidative stress (smoking, outdoor air pollution) in asthma following a candidate gene approach. We cannot exclude that our selection may have overrepresented the antioxidative defense and may have lost a number of relevant genes that were not targeted by our analysis. However, we were able to highlight that genes modulating exposure to LMW agents and irritants all have a prominent role in the NF-κB pathway, and our strategy also has the capacity to generate new hypotheses. One of the difficulties in G × E studies is the need for large studies or consortia to detect significant interaction, which in turn might be affected by heterogeneity in both the outcomes and the exposure definitions of the participating studies. To overcome these limitations, the definition of adult-onset asthma as well as those of occupational exposures to LMW agents or irritants were harmonized across the three epidemiological studies, and genotyping was performed identically in the three studies in the framework of the European Gabriel consortium asthma GWAS (Moffatt et al. 2010). Despite the fact that the three studies were pooled, we obtained 463 exposed participants with adult-onset asthma to detect G × E interactions. This small number of exposed cases may have hampered our findings, and we acknowledge that the lack of replication is a limitation. However, replication is very difficult because EGEA, SAPALDIA, and ECRHS are, to the best of our knowledge, the only three cohorts having such specific information on occupational exposures (the asthma-specific JEM with the expertise step that increases the precision of the exposure assessment and the specific questionnaires for cleaners and health care workers). By adding other studies using only the asthma-specific JEM, we would lose part of the specificity of our analysis. None of our G × E interaction tests reached the significance level after correction for multiple testing, so we focused on SNPs with *p*-values for SNPs × E in the top 1% of the distribution, and we reduced false positives by only selecting consistent interactions across the three studies. With regard to the method used, various study designs and statistical methods have been proposed to investigate G × E interactions (Liu et al. 2012). We used the classical G × E interaction test based on a case–control design, which may not be the most powerful approach. Indeed, when one can assume independence between exposure and SNPs, it has been shown that case-only–based approaches (Mukherjee et al. 2008) are better alternatives. However, these approaches could not be applied to our study because our gene-selection process aimed to select genes potentially associated with the environmental exposure because of their biological function. We further repeated the analyses using a joint test of gene and gene-environment interaction (Dai et al. 2012), but similar results were obtained (data not shown).

Irritant-induced asthma is typically described as a separate, “nonsensitizing,” type of occupational asthma (Maestrelli et al. 2009; Tarlo and Lemiere 2014). However, low molecular weight agents are generally classified as sensitizers, although most of them are not associated with the production of specific immunoglobulin E (IgE) (Tarlo and Lemiere 2014). The precise mechanisms linking irritants and LMW chemicals to asthma are poorly known, and it is therefore challenging to classify most asthmogenic chemicals (e.g., cleaning products) into definite categories. However, occupational exposures to both LMW chemicals and irritants may result in oxidative stress (Dumas et al. 2015). Thus, we could investigate a relatively broad spectrum of exposures by carefully selecting genes through our pathway-based strategy integrating hypotheses about the environment. Smoking is also known to be related to oxidative stress. Our results remained nearly identical after performing analyses excluding current smokers or after accounting for smoking, suggesting that the detected interactions were not due to the effects of smoking.

To our knowledge, none of our findings have been reported previously in published GWASs of asthma (Hindorff et al. 2015), or in GEWIS of asthma. Differences in the lengths of microsatellite sequences in the promoter region of *PLA2G4A* were reported between patients with severe asthma and healthy controls, with a direct impact on mRNA and protein expression, suggesting a role in asthma pathogenesis (Sokolowska et al. 2010). Few candidate G × occupational exposure interaction studies have been published for asthma (Kauffmann et al. 2010; Kogevinas 2014; Smit et al. 2014; Cherry et al. 2015). In a GWAS focusing on occupational exposures, *CTNNA3* (catenin alpha 3, alpha-T catenin) was reported to be the strongest candidate gene for toluene diisocyanate (TDI)-induced asthma in Korean patients (Kim et al. 2009), and only one GEWIS has been published that identified novel susceptibility loci for occupational exposure to biological dust, mineral dust, and gases and fumes in relation to forced expiratory volume in 1 sec (FEV_1_) levels (de Jong et al. 2015).

Interestingly, all of the genes that we detected play a role in the NF-κB pathway. NF-κB is a ubiquitous transcription factor that is involved in the mechanism whereby oxidants affect the pathophysiology of asthma (Schuliga 2015). The genetic variants interacting with exposure do not belong to protein-coding regions; rather, they are more likely to have a regulatory function, as indicated by the functional annotations of a few of these SNPs. *RELA* encodes the RelA protein, which is complexed with NFKB1, the most abundant form of NF-κB. *PRKD1* encodes a serine/threonine kinase called PKD1, which activates NF-κB in response to oxidative stress conditions (Sundram et al. 2011; Storz 2007). Exposure to a photochemically altered air pollutant mixture was associated with a decrease in expression of *PRKD1* mRNA in human lung epithelial cells (Rager et al. 2011). In contrast, exposure to zinc oxide nanoparticles, which is associated with acute pulmonary oxidative stress and inflammation (Vandebriel and De Jong 2012), was reported to activate NF-κB in human bronchial epithelial cells through a mechanism involving RelA–NF-κB phosphorylation (Wu et al. 2010). Interestingly, in a similar manner, we found negative associations between genetic variants of *PRKD1* and adult-onset asthma (decreased risk) and positive associations between genetic variants near *RELA* and adult-onset asthma (increased risk) in participants exposed to LMW or irritant agents. All of these effects are “flip-flop effects,” and we can only speculate on the mechanism behind an opposing effect among the exposed and unexposed subjects. Finally, the protein encoded by *PRKCA* was suggested to be a regulator of NF-κB–induced expression of genes involved in inflammatory responses (Nakashima 2002), and it was associated with generation of reactive oxygen species through a biological interaction with other genes including members of the mammalian PLA2 family (Chi et al. 2014). A role of the secretory phospholipase A2 receptor in the development of asthma was recently reported in animal models of asthma and in human lung cells (Murakami et al. 2014; Leslie 2015). It is noteworthy that the SNPs interacting with exposure identified by this study mapped to binding sites on proteins including NFKB; histone deacetylase 2 (HDAC2), whose activity is regulated by oxidative stress; and nuclear factor erythroid 2-related factor 2 (Nrf2), which plays a crucial role in the cellular defense against oxidative stress. Lastly, chlorine, formaldehyde, and hydrogen peroxide were reported to affect the localization of the *PRKCA* protein, to modify the expression of *PLA2G4A* and *PLA2R1* mRNA, and to modify the activity or expression of *RELA* protein [Comparative Toxicogenomics Database (CTD), http://ctdbase.org/] (Davis et al. 2015). Overall, all these data suggest that any or all of these genes—*PLA2G4A*, *PLA2R1*, *RELA*, *PRKD1*, and *PRKCA*—may play a role in the risk of asthma in adults in association with exposure to LMW agents or irritants.

## Conclusions

In conclusion, the present study identified new and promising candidate genes interacting with occupational exposures to LMW agents or irritants in current adult-onset asthma. More generally, this study highlights the interest in performing G × E interaction analyses to identify new genes and mechanisms of asthma occurrence related to specific environmental exposures.

## Supplemental Material

(533 KB) PDF

(40 KB) ZIP

## Appendix

### Acknowledgments

We thank all study members and staff involved in data collections in each cohort:

***EGEA:*** We thank the Epidemiological Study on Genetics and Environment of Asthma (EGEA) cooperative group members as follows.

**Coordination:** V. Siroux [epidemiology, principal investigator (PI) since 2013]; F. Demenais (genetics); I. Pin (clinical aspects); R. Nadif (biology); F. Kauffmann (PI 1992–2012).

**Respiratory epidemiology:** Inserm U 700, Paris: M. Korobaeff (EGEA1), F. Neukirch (EGEA1); Inserm U 707, Paris: I. Annesi-Maesano (EGEA1–2); Inserm U1168 (ex-CESP/U 1018), Villejuif: F. Kauffmann, N. Le Moual, R. Nadif, M.P. Oryszczyn (EGEA1–2), R. Varraso; Inserm U 823, Grenoble: V. Siroux.

**Genetics:** Inserm U 393, Paris: J. Feingold; Inserm U 946, Paris: E. Bouzigon, F. Demenais, M.H. Dizier; CNG, Evry: I. Gut (now CNAG, Barcelona, Spain), M. Lathrop (now McGill University, Montreal, Canada).

**Clinical centers:** Grenoble: I. Pin, C. Pison; Lyon: D. Ecochard (EGEA1), F. Gormand, Y. Pacheco; Marseille: D. Charpin (EGEA1), D. Vervloet (EGEA1–2); Montpellier: J. Bousquet; Paris Cochin: A. Lockhart (EGEA1), R. Matran (now in Lille); Paris Necker: E. Paty (EGEA1–2), P. Scheinmann (EGEA1–2); Paris Trousseau: A. Grimfeld (EGEA1–2), J. Just.

**Data and quality management:** Inserm ex-U155 (EGEA1): J. Hochez; Inserm U1168 (ex-CESP/U 1018), Villejuif: N. Le Moual; Inserm ex-U780: C. Ravault (EGEA1–2); Inserm ex-U794: N. Chateigner (EGEA1–2); Grenoble: J. Quentin-Ferran (EGEA1–2).

***SAPALDIA:*** We thank the team of the Swiss study on Air Pollution and Lung and Heart Diseases in Adults (SAPALDIA).

**Study directorate:** N.M. Probst-Hensch (PI; e/g); T. Rochat (p), C. Schindler (s), N. Künzli (e/exp), J.M. Gaspoz (c).

**Scientific team:** J.C. Barthélémy (c), W. Berger (g), R. Bettschart (p), A. Bircher (a), C. Brombach (n), P.O. Bridevaux (p), L. Burdet (p), D. Felber Dietrich (e), M. Frey (p), U. Frey (pd), M.W. Gerbase (p), D. Gold (e), E. de Groot (c), W. Karrer (p), F. Kronenberg (g), B. Martin (pa), A. Mehta (e), D. Miedinger (o), M. Pons (p), F. Roche (c), T. Rothe (p), P. Schmid-Grendelmeyer (a), D. Stolz (p), A. Schmidt-Trucksäss (pa), J. Schwartz (e), A. Turk (p), A. von Eckardstein (cc), E. Zemp Stutz (e).

**Scientific team at coordinating centers:** M. Adam (e), I. Aguilera (exp), S. Brunner (s), D. Carballo (c), S. Caviezel (pa), I. Curjuric (e), A. Di Pascale (s), J. Dratva (e), R. Ducret (s), E. Dupuis Lozeron (s), M. Eeftens (exp), I. Eze (e), E. Fischer (g), M. Foraster (e), M. Germond (s), L. Grize (s), S. Hansen (e), A. Hensel (s), M. Imboden (g), A. Ineichen (exp), A. Jeong (g), D. Keidel (s), A. Kumar (g), N. Maire (s), A. Mehta (e), R. Meier (exp), E. Schaffner (s), T. Schikowski (e), M. Tsai (exp).

Abbreviations: (a) allergology, (c) cardiology, (cc) clinical chemistry, (e) epidemiology, (exp) exposure, (g) genetic and molecular biology, (m) meteorology, (n) nutrition, (o) occupational health, (p) pneumology, (pa) physical activity, (pd) pediatrics, (s) statistics.

The study could not have been done without the help of the study participants, technical and administrative support and the medical teams and field workers at the local study sites.

**Local fieldworkers:** Aarau: S. Brun, G. Giger, M. Sperisen, M. Stahel; Basel: C. Bürli, C. Dahler, N. Oertli, I. Harreh, F. Karrer, G. Novicic, N. Wyttenbacher; Davos: A. Saner, P. Senn, R. Winzeler; Geneva: F. Bonfils, B. Blicharz, C. Landolt, J. Rochat; Lugano: S. Boccia, E. Gehrig, M.T. Mandia, G. Solari, B. Viscardi; Montana: A.P. Bieri, C. Darioly, M. Maire; Payerne: F. Ding, P. Danieli, A. Vonnez; Wald: D. Bodmer, E. Hochstrasser, R. Kunz, C. Meier, J. Rakic, U. Schafroth, A. Walder.

**Administrative staff:** N. Bauer Ott, C. Gabriel, R. Gutknecht.

***ECRHS:*** The ECRHS data incorporated in this analysis would not have been available without the collaboration of the following individuals and their research teams.

**ECRHS Co-ordinating Centre.** P. Burney, D. Jarvis, S. Chinn, J. Knox (ECRHS II), C. Luczynska†, J. Potts.

**Steering Committee for ECRHS II.** P. Burney, D. Jarvis, S. Chinn, U. Ackermann-Liebrich, J.M. Anto, I. Cerveri, R. deMarco†, T. Gislason, J. Heinrich, C. Janson, N. Kunzli, B. Leynaert, F. Neukirch, J. Schouten, J. Sunyer; C. Svanes, P. Vermeire†, M. Wjst.

**Principal Investigators and Senior Scientific Teams for ECRHS II centers within this analysis:** Estonia: Tartu (R. Jogi, A. Soon); France: Paris (F. Neukirch, B. Leynaert, R. Liard, M. Zureik), Grenoble (I. Pin, J. Ferran-Quentin); Germany: Erfurt (J. Heinrich, M. Wjst, C. Frye, I. Meyer), Hamburg (K. Richter, D. Nowak); Norway: Bergen (A. Gulsvik, E. Omenaas, C. Svanes, B. Laerum); Spain: Barcelona (J.M. Anto, J. Sunyer, M. Kogevinas, J.P. Zock, X. Basagana, A. Jaen, F. Burgos), Huelva (J. Maldonado, A. Pereira, J.L. Sanchez), Albacete (J. Martinez-Moratalla Rovira, E. Almar), Galdakao (N. Muniozguren, I. Urritia), Oviedo (F. Payo); Sweden: Uppsala (C. Janson, G. Boman, D. Norback, M. Gunnbjornsdottir), Umeå (E. Norrman, M. Soderberg, K. Franklin, B. Lundback, B. Forsberg, L. Nystrom); Switzerland: Basel (N. Kunzli, B. Dibbert, M. Hazenkamp, M. Brutsche, U. Ackermann-Liebrich); United Kingdom: Norwich (D. Jarvis, B. Harrison), Ipswich (D. Jarvis, R. Hall, D. Seaton).

†Deceased.

### Funding Information

The genotyping of all three studies was funded by the French National Agency of Research (ANR-PRSP 2009: IAGO), and by the European Commission (contract no. 018996) (GABRIEL) and the Wellcome Trust grant (WT 084703MA), both awarded to the GABRIEL consortium (a multidisciplinary study to identify the genetic and environmental causes of asthma in the European Community).

***EGEA:*** Research funded by the French Agency of Health Safety, Environment and Work (AFSSET, EST-09-15).

***SAPALDIA:*** The Swiss National Science Foundation (grants 33CS30-148470/1, 33CSCO-134276/1, 33CSCO-108796, 324730_135673, 3247BO-104283, 3247BO-104288, 3247BO-104284, 3247-065896, 3100-059302, 3200-052720, 3200-042532, 4026-028099, PMPDP3_129021/1, PMPDP3_141671/1), the Federal Office for the Environment, the Federal Office of Public Health, the Federal Office of Roads and Transport, the canton governments of Aargau, Basel-Stadt, Basel-Land, Geneva, Luzern, Ticino, Valais, and Zürich, the Swiss Lung League, the canton Lung Leagues of Basel Stadt/Basel Landschaft, Geneva, Ticino, Valais, Graubünden and Zurich, Stiftung ehemals Bündner Heilstätten, Swiss National Accident Insurance Fund (SUVA), Freiwillige Akademische Gesellschaft, UBS Wealth Foundation, Talecris Biotherapeutics GmbH, Abbott Diagnostics.

***ECRHS:*** The coordination of ECRHS II was supported by the European Commission as part of their Quality of Life program. This work was also funded by the U.S. National Institutes of Health (NIH; grant 1R01HL062633) and the Carlos III Health Institute of the Spanish Ministry of Health and Consumption (FIS; grant 01/3058).

The following bodies funded the local studies in ECRHS II included in this paper: **Albacete:** Fondo de Investigaciones Santarias (FIS) (grants 97/0035-01, 99/0034-01, 99/0034-02), Hospital Universitario de Albacete, Consejeria de Sanidad; **Barcelona:** SEPAR, Public Health Service (grant R01 HL62633-01), FIS (grants 97/0035-01, 99/0034-01, 99/0034-02), CIRIT (grant 1999SGR 00241), Red Respira ISCII; **Basel:** Swiss National Science Foundation, Swiss Federal Office for Education and Science, Swiss National Accident Insurance Fund (SUVA), University of Southern California (USC) NIEHS Center grant 5P30 ES07048; **Bergen:** Norwegian Research Council, Norwegian Asthma and Allergy Association (NAAF), Glaxo Wellcome AS, Norway Research Fund; **Erfurt:** GSF-National Research Centre for Environment and Health, Deutsche Forschungsgemeinschaft (DFG) (grant FR 1526/1-1); **Galdakao:** Basque Health Dept; **Grenoble:** Programme Hospitalier de Recherche Clinique-DRC de Grenoble 2000 no. 2610, Ministry of Health, Direction de la Recherche Clinique, Ministere de l’Emploi et de la Solidarite, Direction Generale de la Sante, CHU de Grenoble, Comite des Maladies Respiratoires de l’Isere; **Hamburg:** GSF-National Reasearch Centre for Environment and Health, Deutsche Forschungsgemeinschaft (DFG) (grant MA 711/4-1); **Ipswich and Norwich:** Asthma UK (formerly known as National Asthma Campaign); **Huelva:** Fondo de Investigaciones Santarias (FIS) (grants 97/0035-01, 99/0034-01, 99/0034-02); **Oviedo:** Fondo de Investigaciones Santarias (FIS) (grants 97/0035-01, 99/0034-01 99/0034-02); **Paris:** Ministere de l’Emploi et de la Solidarite, Direction Generale de la Santé, UCB-Pharma (France), Aventis (France), Glaxo France, Programme Hospitalier de Recherche Clinique-DRC de Grenoble 2000 no. 2610, Ministry of Health, Direction de la Recherche Clinique, CHU de Grenoble; **Tartu:** Estonian Science Foundation; **Umeå:** Swedish Heart Lung Foundation, Swedish Foundation for Health Care Sciences and Allergy Research, Swedish Asthma and Allergy Foundation, Swedish Cancer and Allergy Foundation; **Uppsala:** Swedish Heart Lung Foundation, Swedish Foundation for Health Care Sciences and Allergy Research, Swedish Asthma and Allergy Foundation, Swedish Cancer and Allergy Foundation.
