# Supplementary material for: Genes Interacting with Occupational Exposures to Low Molecular Weight Agents and Irritants on Adult-Onset Asthma in Three European Studies
Source: Environ Health Perspect. 2016 Aug 9;125(2):207–14. doi: 10.1289/EHP376 (PMC5289825; doi:10.1289/EHP376)
Supplement: (533 KB) PDF [file EHP376.s001.acco.pdf]

Environ Health Perspect
DOI: 10.1289/EHP376

Note to readers with disabilities: *EHP* strives to ensure that all journal content is accessible to all readers. However, some figures and Supplemental Material published in *EHP* articles may not conform to 508 standards due to the complexity of the information being presented. If you need assistance accessing journal content, please contact ehp508@niehs.nih.gov. Our staff will work with you to assess and meet your accessibility needs within 3 working days.

Supplemental Material

Genes Interacting with Occupational Exposures to Low Molecular Weight Agents and Irritants on Adult-Onset Asthma in Three European Studies

Marta Rava, Ismail Ahmed, Manolis Kogevinas, Nicole Le Moual, Emmanuelle Bouzigon, Ivan Curjuric, Marie-Hélène Dizier, Orianne Dumas, Juan R. Gonzalez, Medea Imboden, Amar J. Mehta, Pascale Tubert-Bitter, Jan-Paul Zock, Deborah Jarvis, Nicole M. Probst-Hensch, Florence Demenais, and Rachel Nadif

Table of Contents

Description of the three multicentre epidemiological European studies

The EGEA study

The ECRHS study

The SAPALDIA study

REFERENCES

Table S1. Assessment of occupational exposure and definition of exposed and non-exposed groups

Table S2. Description of SNPs interacting at the P<0.005 level with occupational exposure to LMW agents or irritants on current adult-onset asthma (463 cases/2136 controls)

Table S3: Interactions between SNPs and occupational exposure to LMW agents or irritants on current adult-onset asthma stratified by study.

Table S4: Interactions between SNPs in *PLAG24A*, *RELA*, *PRKD1* and *PRKCA* and occupational exposure to LMW agents or irritants on current adult-onset asthma after accounting for family dependency, after excluding current smokers, and after further adjustment for smoking status (case-control approach)

Table S5. Gene-based interaction results obtained with VEGAS at the P-value<.05.

Figure S1. Flow-charts of the selected participants in each European study (**A**: EGEA, **B**: SAPALDIA and **C**: ECRHS).

Figure S2. Summary of the SNP-occupational exposure interaction results around rs932476 in *PLA2G4A* (**A**), rs2667026 in *PLA2R1* (**B**), rs931127 in *RELA*(**C**) , rs1958980 in *PRKD1* (**D**) and rs6504453 in *PRKCA* (**E**) using the LocusZoom software (Pruim et al. 2010). The left Y-axis shows the -log10 P-values. The position of the SNPs in megabases (build 36.3) is shown on the X-axis. rs932476, rs2667026, rs931127, rs1958980 and rs6504453 are colored in purple. The degree of LD (from the hg/1000 Genomes reference panel) between these SNPs and the SNPs we selected in the same region is reflected by the color of the dots (red being the highest degree of LD). The blue line represents the recombination rate (right Y-axis).

Figure S3. Association between T allele at rs6504453 in *PRKCA* and gene expression in lung tissue (eQTL browser GTEx, Gibson et al. 2015).

Additional Files

- **Supplemental Code and Data ZIP File**
  - **Supplemental Code and Data ZIP File Index**
  - **Excel File Tables S1-S5**
    - **Excel File Table S1.** Canonical pathways involved in the metabolism of LMW agents or irritants
    - **Excel File Table S2.** List of the 163 genes selected using the candidate pathway-based strategy

Excel File Table S3. List of the 17 pathways in which the four genes are involved among the other relevant genes selected by the candidate pathway-based strategy

Excel File Table S4. Regulatory elements of the four loci (1q31.1, 2q24.2, 11q13.1 and 14q12) containing SNPs in bold (rs932476, rs2667026, rs931127, rs7949980, rs1958980, rs11847351, and rs1958987) interacting with occupational exposures to LMW agents or irritants on current adult-onset asthma. Functional annotation of these SNPs (or SNPs in high LD (D'>0.9; r2>=0.8) was searched using HaploReg (http://www.broadinstitute.org/mammals/haploreg/haploreg.php , Ward and Kellis, 2012). In this table are indicated the cell lines in which elements are present (enhancer histone marks and DNase hypersensitivity sites) or regulatory motifs or the type protein that binds to this specific site (Proteins bound).

Excel File Table S5. Chemical retrieved from the Comparative Toxicogenomics Database (CTD, URL: http://ctdbase.org/, Davis et al. 2014) and interacting with the five genes (PLA2G4A, PLA2R1, PRKCA, PRKD1 and RELA). In this table are indicated the LMW agents/irritants/cleaning products or disinfectants evaluated by job specific questionnaires (see Table E1) or exposures known to contain compounds with irritant properties (air pollutants and vehicle emissions).

Description of the three multicentre epidemiological European studies

The EGEA study

The EGEA study is a 12-year longitudinal survey, which combines a case-control study and a family study of asthmatic cases (https://egeanet.vjf.inserm.fr/index.php/en/). The protocol and descriptive characteristics have been described elsewhere (Kauffmann et al. 1997, 1999). Probands (388 asthmatic patients) were recruited from six chest clinics in five French cities between 1991 and 1995 and their 1,244 family members were included, either by including the proband's parents and siblings, or by including the proband's spouse and children. In addition, 415 population-based controls were recruited. The probands (asthmatics and controls) were between 7 and 70 years old at time of ascertainment. All probands and their two parents were of European ancestry and were born in France. A follow-up (EGEA2) of the initial cohort was conducted between 2003 and 2007 and included follow-up data for 1,543 participants from the initial cohort and 73 new family members. All participants responded to a questionnaire based on international standardized tools to diagnose asthma and to determine respiratory and allergic symptoms, treatments, and environmental and occupational exposures (Kauffmann et al. 1997). As a follow-up study of EGEA2, the third survey (EGEA3) was conducted in 2011 using self-completed questionnaire and 1558 questionnaires were returned (Bouzigon et al. 2015).

The present analysis uses data measured at the second stage of the study (EGEA2) in 689 adult participants with complete data with data available on genome-wide genotyping, occupational history regarding LMW agents and irritants, especially cleaning/disinfecting products, and adult-onset asthma (Figure S1). Ethical approval was obtained from the relevant institutional review board committees (Cochin Port-Royal Hospital and Necker- Enfants Malades Hospital, Paris). Written informed consent was signed by all participants.

The ECRHS study

ECRHS is a random population-based multicenter cohort study of participants aged 20–44 years at the time of recruitment (1991-93; ECRHS I). The follow-up (ECRHS II) took place approximately 9 years later (median length of follow-up, 8.9 years), during the period 2000-2002. Both surveys included an initial screening questionnaire, an extensive interviewer-led questionnaire, spirometry and a methacholine challenge test. For further details see (Burney et al. 1994; "The European Community Respiratory Health Survey II" 2002).

The SAPALDIA study

The SAPALDIA prospective population-based cohort study (Ackermann-Liebrich et al. 2005; Downs et al. 2007) recruited a random sample of inhabitants aged 18–60 years from eight areas of Switzerland representing various degrees of urbanization and air pollution exposure. Participants underwent spirometry as well as a detailed interview on respiratory health, smoking history, lifestyle factors and anthropometry at both, baseline (1991) and follow-up examination (2002). From among 6,055 SAPALDIA cohort participants participating in both examinations and agreeing to provide blood for genetic analysis, all self-reported asthma cases as well as a random sample of controls had genome wide genotyping in the framework of the European funded "GABRIEL" asthma project (Moffat et al. 2010). For the purposes of the current gene-environment interaction analysis, the case definition was restricted to cases of adult asthma (age of onset larger than 16 years) with current treatment or symptoms within the last 12 months prior to assessment. For the definition of occupational risk exposures, job history during the period between SAPALDIA-1 and SAPALDIA-2, and/or reported at the time of SAPALDIA-1 was considered. The genome wide-data set formed the basis for the candidate-pathway based genetic

analysis. Overall, n=107 asthma cases and n=467 controls were included in the current pooled analysis.

REFERENCES

Ackermann-Liebrich U, Kuna-Dibbert B, Probst-Hensch NM, Schindler C, Felber Dietrich D, Stutz EZ, et al. 2005. Follow-up of the Swiss Cohort Study on Air Pollution and Lung Diseases in Adults (SAPALDIA 2) 1991-2003: methods and characterization of participants. Soz Praventivmed 50:245-263.

Bouzigon E, Nadif R, Le Moual N, Dizier MH, Aschard H, Boudier A, et al. 2015. [Genetic and environmental factors of asthma and allergy: Results of the EGEA study]. Rev Mal Respir 32:822-840.

Burney PG, Luczynska C, Chinn S, Jarvis D. 1994. The European Community Respiratory Health Survey. Eur Respir J 7:954-960.Downs SH, Schindler C, Liu LJ, Keidel D, Bayer-Oglesby L, Brutsche MH, et al. 2007. Reduced exposure to PM10 and attenuated age-related decline in lung function. N Engl J Med 357:2338–2347.

Downs SH, Schindler C, Liu LJ, Keidel D, Bayer-Oglesby L, Brutsche MH, et al. 2007. Reduced exposure to PM10 and attenuated age-related decline in lung function. N Engl J Med 357:2338–2347.

European Community Respiratory Health Survey II Steering Committee. 2002. The European Community Respiratory Health Survey II. Eur Respir J 20:1071–1079.

Gibson G. 2015. Human genetics. GTEx detects genetic effects. Science 348:640-641.

Kauffmann F, Dizier MH, Annesi-Maesano I, Bousquet J, Charpin D, Demenais F, et al. 1999. EGEA (Epidemiological study on the Genetics and Environment of Asthma, bronchial

hyperresponsiveness and atopy)-- descriptive characteristics. Clin Exp Allergy 29 Suppl 4: 17-21.

Kauffmann F, Dizier MH, Pin I, Paty E, Gormand F, Vervloet D, et al. 1997. Epidemiological study of the genetics and environment of asthma, bronchial hyperresponsiveness, and atopy: phenotype issues. Am J Respir Crit Care Med 156:S123-S129.

Moffatt MF, Gut IG, Demenais F, et al. A large-scale, consortium-based genomewide association study of asthma. N Engl J Med 2010; 363: 1211–1221.

Pruim RJ, Welch RP, Sanna S, Teslovich TM, Chines PS, Gliedt TP, et al. 2010. LocusZoom: regional visualization of genome-wide association scan results. Bioinformatics 26:2336-2337.

Table S1. Assessment of occupational exposure and definition of exposed and non-exposed groups

**Exposed**	**Exposures to asthmagenic agents (ever) evaluated by the asthma-specific JEM:**<br>**LMW agents**: Highly reactive chemicals, cleaning/disinfecting products, metals antigenic, wood dust, isocyanates<br>**Mixed environment with potential exposure to LMW agents**: textile production, agriculture, metal working fluids exposures, pharmaceutical drugs exposures, bioaerosol exposures<br>**Irritants**: accidental peak exposure	**OR**	**Exposures to cleaning products and disinfectants (ever) evaluated by job specific questionnaires** (self-reported weekly exposure):<br>**Cleaners**: bleach, ammonia, decalcifiers, solvents, formaldehyde, alcohol, quaternary ammonium compounds, and disinfection tasks<br>**Healthcare workers**: bleach, ammonia, decalcifiers, solvents, formaldehyde, glutaraldehyde, alcohol, quaternary ammonium compounds, chloramine T, ethylene oxide, and disinfection tasks
**Excluded***	**Exposures evaluated by the asthma JEM:**<br>**HMW agents**, inducing asthma through typical allergic (IgE-mediated) response<br>Jobs with **lower level of irritant** exposures	**OR**	**Exposures evaluated by the ALOHA JEM:**<br>General dust, gases and fumes
**Unexposed group (reference)**	**Non exposed to any agent** potentially a risk for asthma or respiratory health		
JEM – Job Exposure Matrix; HMW: High Molecular Weight; LMW – Low Molecular Weight<br>* In order to increase specificity of both exposed and unexposed groups			

Table S2. Description of SNPs interacting at the P<0.005 level with occupational exposure to LMW agents or irritants on current adult-onset asthma (463 cases/2136 controls)

**Chr**	**Gene**	**Region**	**SNP**	**Position** [a]	**Ref./Effect allele**	**EAF** [b]	**Marginal effect OR/P-value**		**Interaction - CC OR/P-value**		**Test for heterogeneity**		
											**I_2^c**	**chi-2**	**P-value**
**1**	*PRDX6*	/	rs10912626	171739312	A/G	0.21	1.06	0.5415	0.55	0.0014	71.5	7.67	0.02
1	*PRDX6*	/	rs6687872	171740885	A/G	0.79	0.95	0.5532	1.82	0.0014	71.5	7.66	0.02
1	*PLA2G4A*	Intronic	rs932476	185200178	A/G	0.35	1.25	**0.0036**	0.64	0.0050	**0.0**	**1.45**	**0.48**
**2**	*PLA2R1*	2.4 kb in 5'	rs2667026	160629801	A/G	0.83	0.89	0.2354	1.77	0.0050	**0.0**	**2.27**	**0.32**
**8**	*CLU*	/	rs569214	27543709	G/T	0.39	0.97	0.7048	0.58	0.0006	61.2	4.94	0.08
**10**	*CDK1*	3' UTR	rs10711	62224480	G/T	0.69	1.08	0.3405	1.63	0.0041	54.0	4.40	0.11
**11**	*RELA*	/	rs931127	65161876	A/G	0.43	1.17	**0.0350**	1.61	0.0014	**0.0**	**0.22**	**0.89**
11	*RELA*	15kb in 5'	rs7949980	65202526	C/T	0.51	1.07	0.3421	1.56	0.0030	**0.0**	**0.01**	**0.99**
**14**	*PRKD1*	Intronic	rs7157367	29375612	A/G	0.33	0.92	0.2926	1.59	0.0033	27.3	2.67	0.26

14	*PRKD1*	Intronic	rs1958980	29381842	A/G	0.67	1.08	0.3344	0.64	0.0042	**20.9**	**2.40**	**0.30**
14	*PRKD1*	Intronic	rs11847351	29383829	A/G	0.67	1.08	0.3429	0.64	0.0043	**21.9**	**2.43**	**0.30**
14	*PRKD1*	Intronic	rs1958987	29409562	C/T	0.68	1.07	0.3609	0.64	0.0054	**0.0**	**0.17**	**0.92**
**17**	*PRKCA*	Intronic	rs6504453	62166692	C/T	0.35	1.04	0.6086	0.63	0.0032	**0.0**	**1.00**	**0.61**
**20**	*PLCB1*	Intronic	rs227142	8367397	C/T	0.11	1.08	0.4852	1.82	0.0050	**74.7**	**8.74**	**0.01**

[a]Position in base pairs (bp), hg18. [b] Effect Allele Frequency (EAF) calculated in controls. [c] Proportion of between-study variability; **0%-24%**: little heterogeneity, 25%-49%: moderate heterogeneity, 50%-74%: large heterogeneity, and >75%: very large heterogeneity.
Chr: chromosome, CC: case-control.

Table S3: Interactions between SNPs and occupational exposure to LMW agents or irritants on current adult-onset asthma stratified by study.

				EGEA (122 cases/567 controls)			SAPALDIA (107 cases/467 controls)			ECRHS (234 cases/1102 controls)		
**Chr**	**Gene**	**SNP**	**Reference / Effect allele**	**EAF [a]**	**OR ± SE**	**P-value**	**EAF [a]**	**OR ± SE**	**P-value**	**EAF [a]**	**OR ± SE**	**P-value**
**1**	*PLA2G4A*	rs932476	A/G	0.34	0.72 ± 0.31	0.29	0.35	0.44 ± 0.37	0.02	0.34	0.71 ± 0.23	0.13
2	*PLA2R1*	rs2667026	A/G	0.82	2.76 ± 0.41	0.01	0.82	1.11 ± 0.47	0.82	0.84	1.58 ± 0.28	0.10
**11**	*RELA*	rs931127	A/G	0.43	1.86 ± 0.29	0.03	0.42	1.70 ± 0.33	0.11	0.44	1.57 ± 0.21	0.03
11	*RELA*	rs7949980	C/T	0.51	1.65 ± 0.28	0.08	0.50	1.63 ± 0.34	0.15	0.51	1.59 ± 0.21	0.03
**14**	*PRKD1*	rs1958980	A/G	0.67	0.55 ± 0.31	0.05	0.64	0.41 ± 0.36	0.01	0.68	0.77 ± 0.22	0.23
14	*PRKD1*	rs11847351	A/G	0.67	0.55 ± 0.31	0.05	0.64	0.41 ± 0.36	0.01	0.68	0.77 ± 0.22	0.24
14	*PRKD1*	rs1958987	C/T	0.67	0.66 ± 0.30	0.18	0.65	0.61 ± 0.35	0.18	0.69	0.56 ± 0.22	0.01
**17**	*PRKCA*	rs6504453	C/T	0.35	0.48 ± 0.31	0.02	0.36	0.67 ± 0.36	0.26	0.35	0.70 ± 0.22	0.11

[a] Effect Allele Frequency (EAF) calculated in controls

Table S4: Interactions between SNPs in *PLAG24A*, *RELA*, *PRKD1* and *PRKCA* and occupational exposure to LMW agents or irritants on current adult-onset asthma after accounting for family dependency, after excluding current smokers, and after further adjustment for smoking status (case-control approach)

**Chr**	**Gene**	**SNP**	**Reference /Effect Allele**	**Interaction (n=2599) - GLM**		**Results accounting for family dependency (n=2599) - GEE**		**Results after excluding current smokers (n=1902) - GLM**		**Results adjusted for smoking (n=2599) - GLM**	
				**OR ± SE**	P-value	**OR ± SE**	P-value	**OR ± SE**	P-value	**OR ± SE**	P-value
**1**	*PLA2G4A*	rs932476	A/G	0.64 ± 0.16	0.0055	0.64 ± 0.16	0.0048	0.77 ± 0.18	0.16	0.64 ± 0.16	0.0057
**2**	*PLA2R1*	rs2667026	A/G	1.77 ± 0.20	0.0050	1.77 ± 0.21	0.0066	1.64 ± 0.23	0.03	1.76 ± 0.20	0.0057
**11**	*RELA*	rs931127	A/G	1.61 ± 0.15	0.0015	1.61 ± 0.14	0.0010	1.74 ± 0.17	0.0013	1.63 ± 0.15	0.0012
**11**	*RELA*	rs7949980	C/T	1.56 ± 0.15	0.0030	1.56 ± 0.15	0.0029	1.64 ± 0.18	0.0045	1.56 ± 0.15	0.0032
**14**	*PRKD1*	rs1958980	A/G	0.64 ± 0.16	0.0042	0.63 ± 0.16	0.0038	0.68 ± 0.18	0.04	0.64 ± 0.16	0.0054
**14**	*PRKD1*	rs11847351	A/G	0.64 ± 0.16	0.0043	0.63 ± 0.16	0.0039	0.69 ± 0.18	0.04	0.64 ± 0.16	0.0055
**14**	*PRKD1*	rs1958987	C/T	0.64 ± 0.16	0.0054	0.64 ± 0.16	0.0042	0.67 ± 0.19	0.03	0.64 ± 0.16	0.0059
**17**	*PRKCA*	rs6504453	C/T	0.63 ± 0.16	0.0032	0.63 ± 0.16	0.0036	0.58 ± 0.19	0.0036	0.62 ± 0.16	0.0028

Chr: chromosome, CC: case-control.

Table S5. Gene-based interaction results obtained with VEGAS at the P-value<.05.

Chr	Gene	Number of SNPs	Start Position	Stop Position	Test	P-value
**11**	***RELA***	6	65178392	65186951	28.5	**0.009**
14	*FOS*	9	74815283	74818665	23.4	0.024
1	*PRDX6*	8	171713108	171724569	26.8	0.030
10	*MGMT*	71	131155455	131455358	146.7	0.030
22	*GSTT2B*	1	22629600	22633368	4.4	0.035
**6**	*ARG1*	5	131936057	131947161	14.8	0.036
**14**	***PRKD1***	50	29115437	29466650	109.9	**0.036**
22	*NDUFA6*	4	40811475	40816834	13.8	0.047

Chr: chromosome.

Figure S1A. Flow-chart EGEA

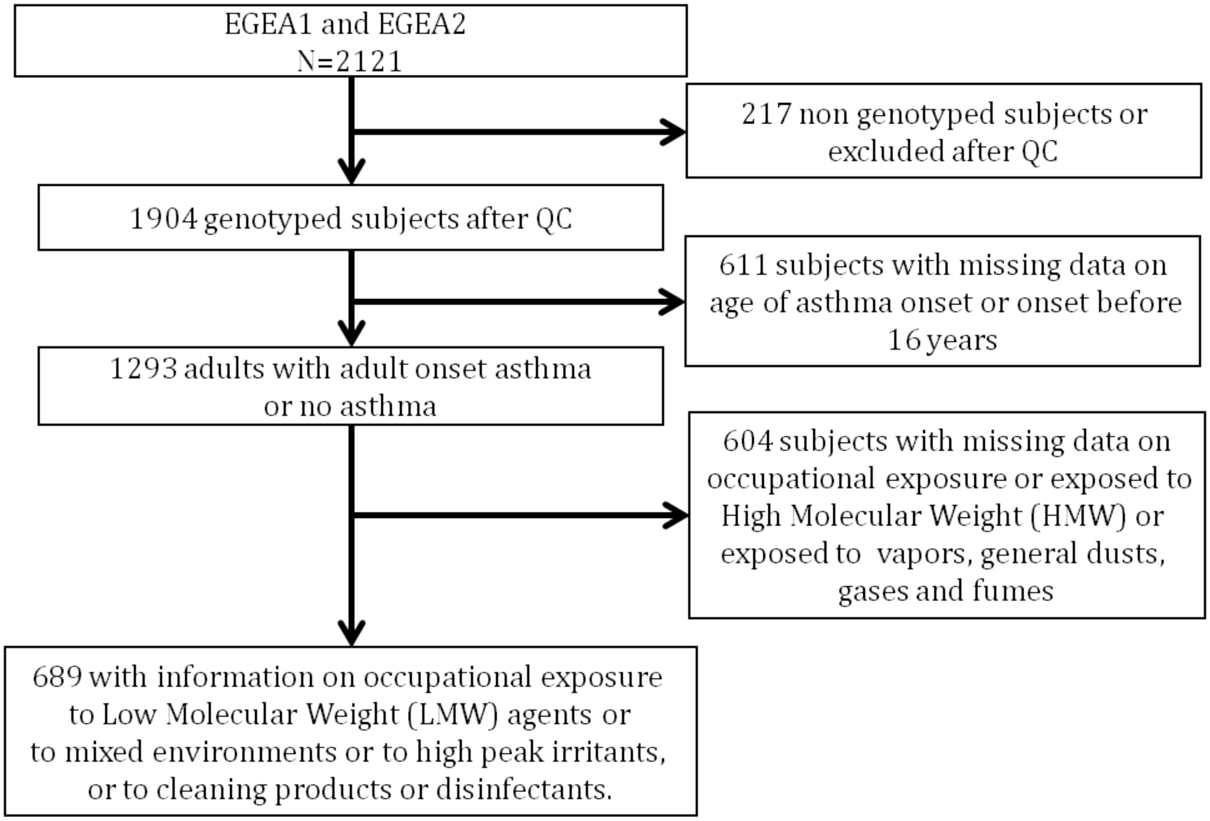

Figure S1B. Flow-chart SAPALDIA

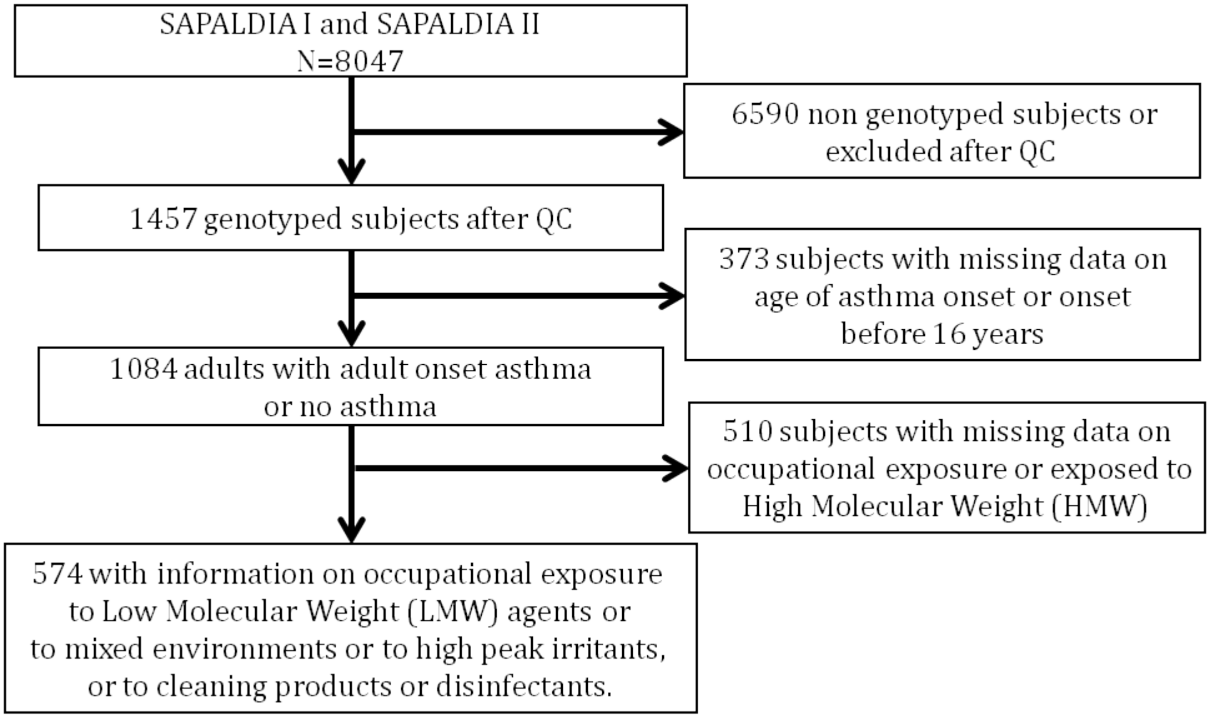

Figure S1C. Flow-chart ECRHS

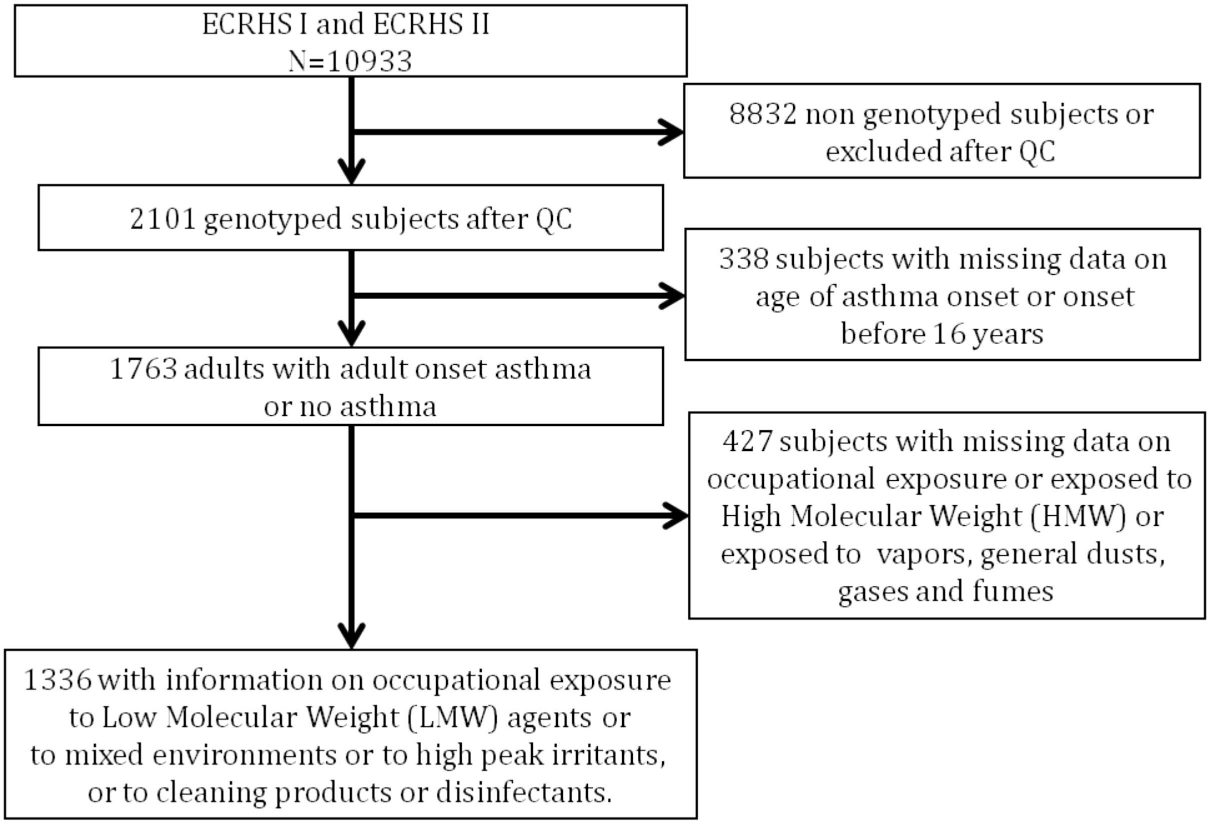

Figure S1. Flow-charts of the selected participants in each European study (**A**: EGEA, **B**: SAPALDIA and **C**: ECRHS).

Figure S2A

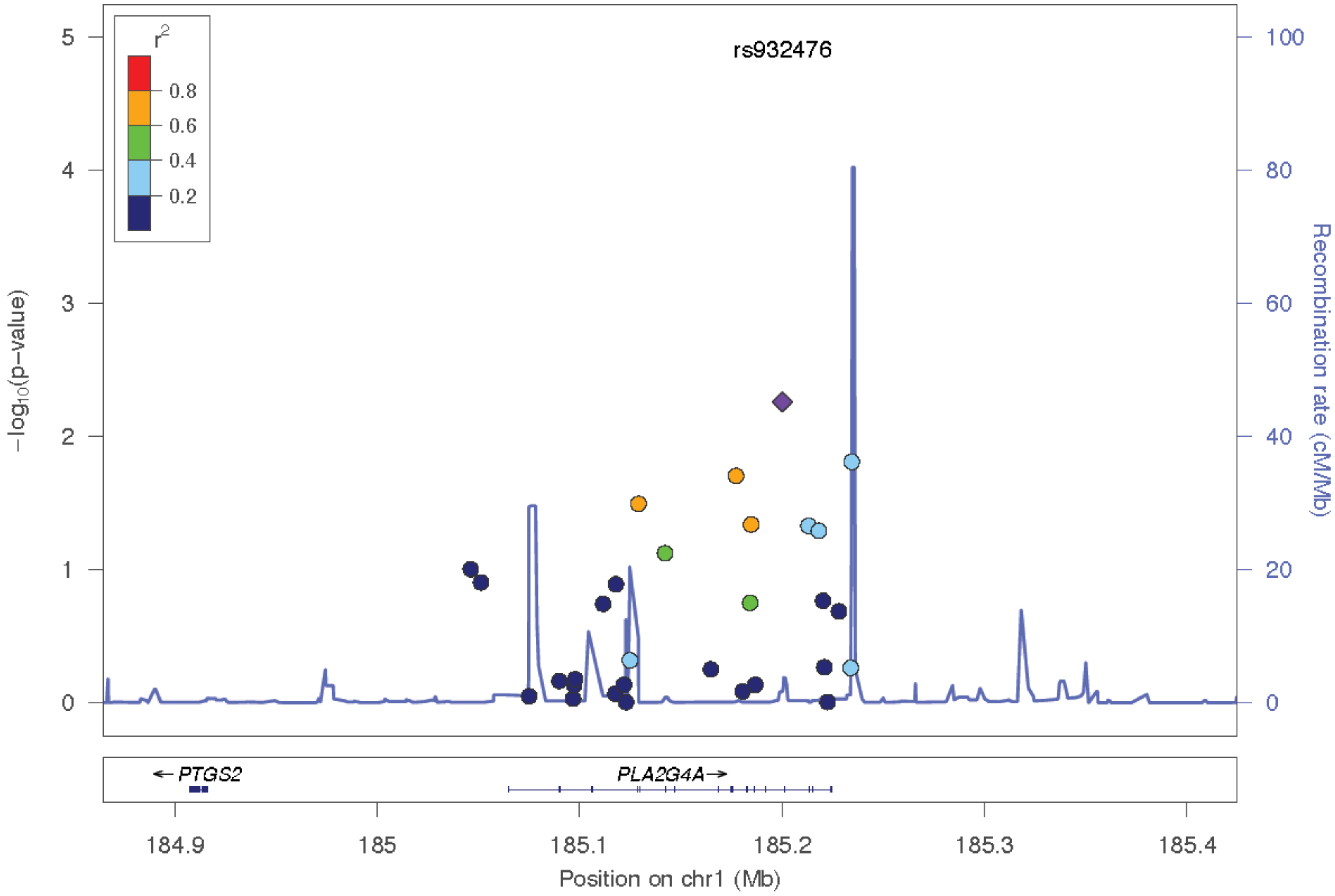

rs932476
r2
0.8
0.6
0.4
0.2
−log10(p−value)
Recombination rate (cM/Mb)
← PTGS2
PLA2G4A →
184.9
185
185.1
185.2
185.3
185.4
Position on chr1 (Mb)

Figure S2B

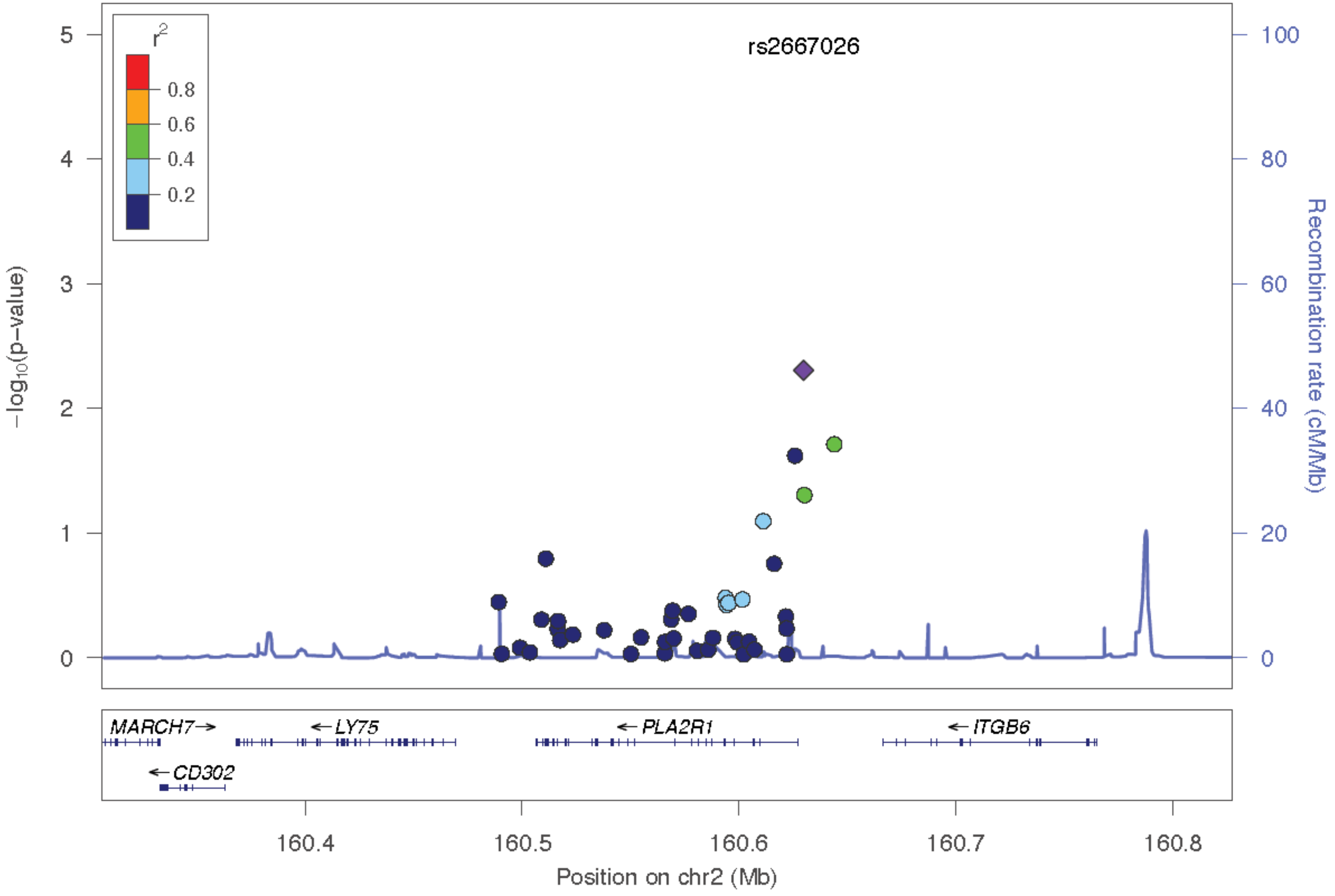

rs2667026
r²
0.8
0.6
0.4
0.2
5
4
3
2
1
0
−log10(p−value)
100
80
60
40
20
0
Recombination rate (cM/Mb)
MARCH7→
←LY75
←PLA2R1
←ITGB6
←CD302
160.4
160.5
160.6
160.7
160.8
Position on chr2 (Mb)

Figure S2C

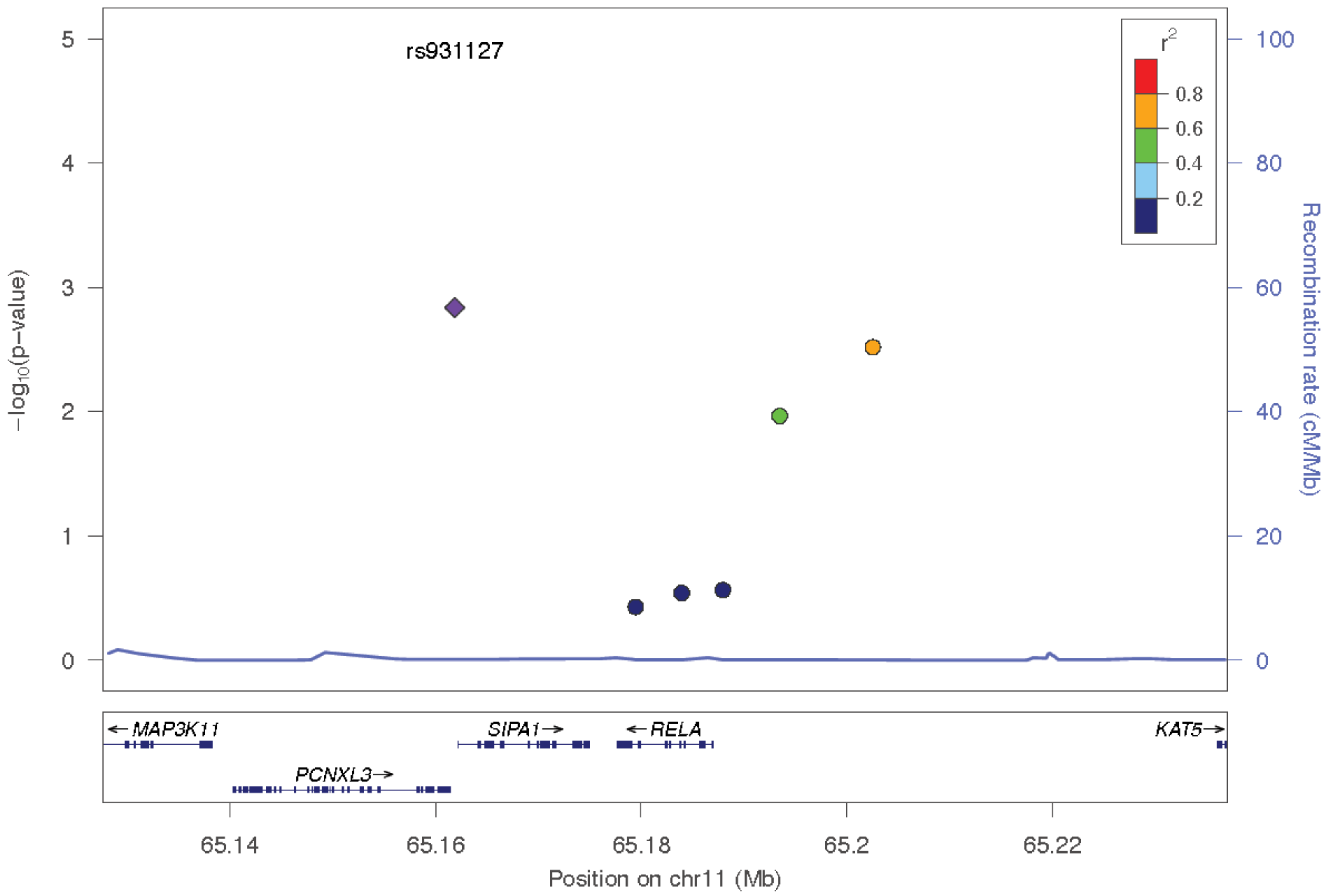
rs931127
r2
0.8
0.6
0.4
0.2
5
4
3
2
1
0
−log10(p−value)
100
80
60
40
20
0
Recombination rate (cM/Mb)
MAP3K11
PCNXL3
SIPA1
RELA
KAT5
65.14
65.16
65.18
65.2
65.22
Position on chr11 (Mb)

Figure S2D

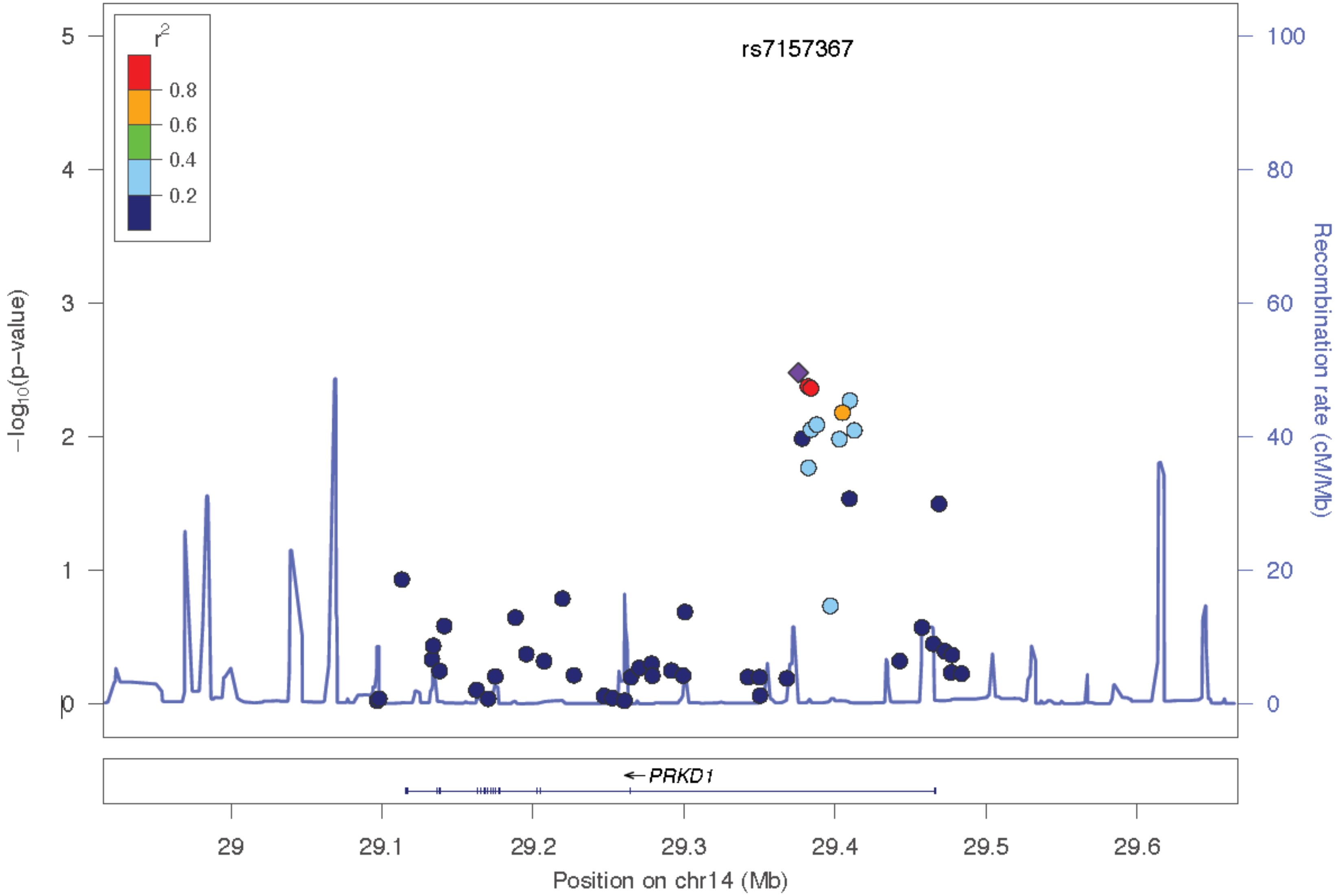

rs7157367
r²
0.8
0.6
0.4
0.2
−log10(p−value)
Recombination rate (cM/Mb)
PRKD1
Position on chr14 (Mb)
29
29.1
29.2
29.3
29.4
29.5
29.6
0
1
2
3
4
5
20
40
60
80
100

Figure S2E

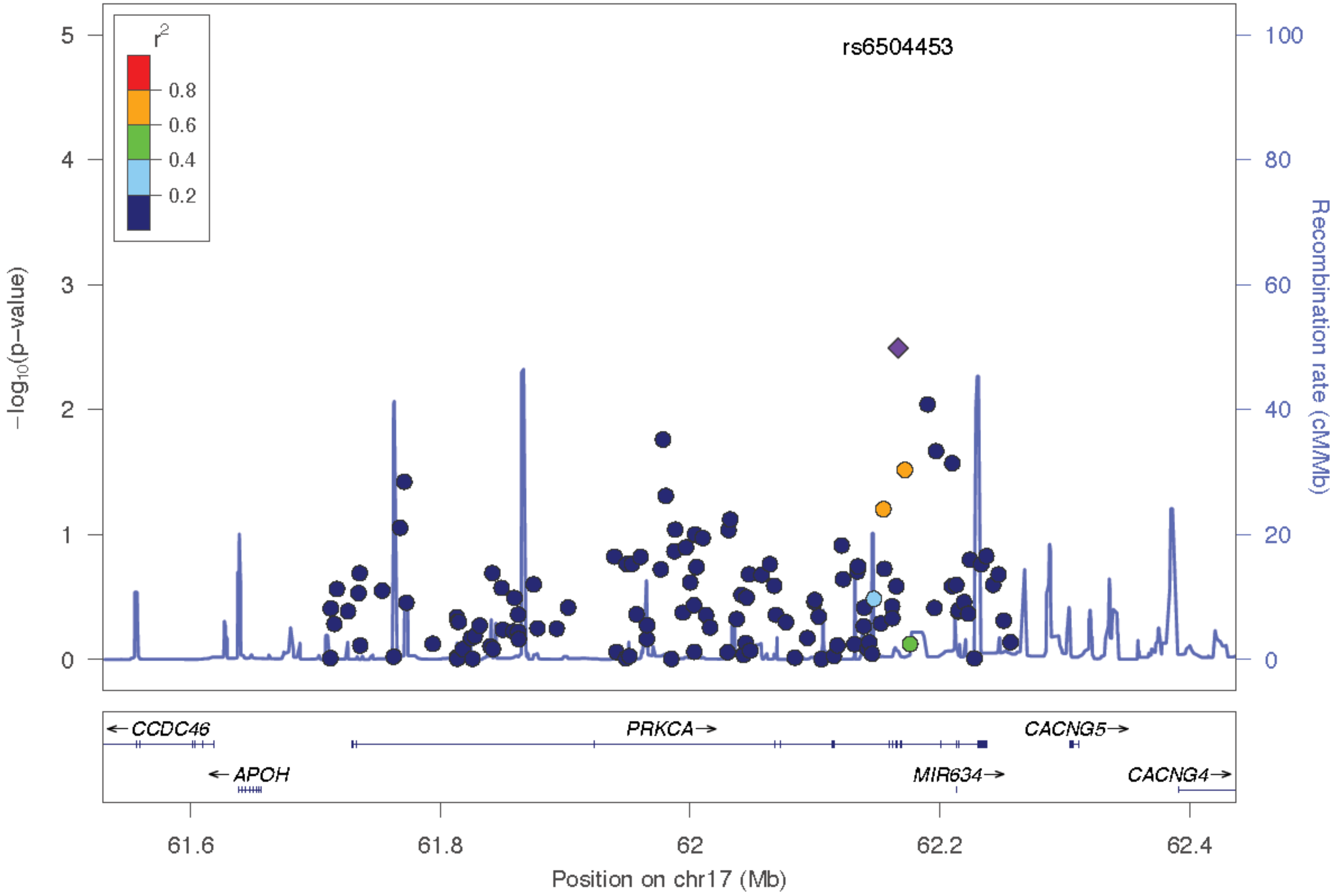

r²
0.8
0.6
0.4
0.2
rs6504453
−log10(p−value)
Recombination rate (cM/Mb)
5
4
3
2
1
0
100
80
60
40
20
0
← CCDC46
PRKCA→
CACNG5→
← APOH
MIR634→
CACNG4→
61.6
61.8
62
62.2
62.4
Position on chr17 (Mb)

Figure S2. Summary of the SNP-occupational exposure interaction results around rs932476 in *PLA2G4A* (**A**), rs2667026 in *PLA2R1* (**B**), rs931127 in *RELA*(**C**) , rs1958980 in *PRKD1* (**D**) and rs6504453 in *PRKCA* (**E**) using the LocusZoom software (Pruim et al. 2010).

The left Y-axis shows the -log10 P-values. The position of the SNPs in megabases (build 36.3) is shown on the X-axis. rs932476, rs2667026, rs931127, rs1958980 and rs6504453 are colored in purple. The degree of LD (from the hg/1000 Genomes reference panel) between these SNPs and the SNPs we selected in the same region is reflected by the color of the dots (red being the highest degree of LD). The blue line represents the recombination rate (right Y-axis).

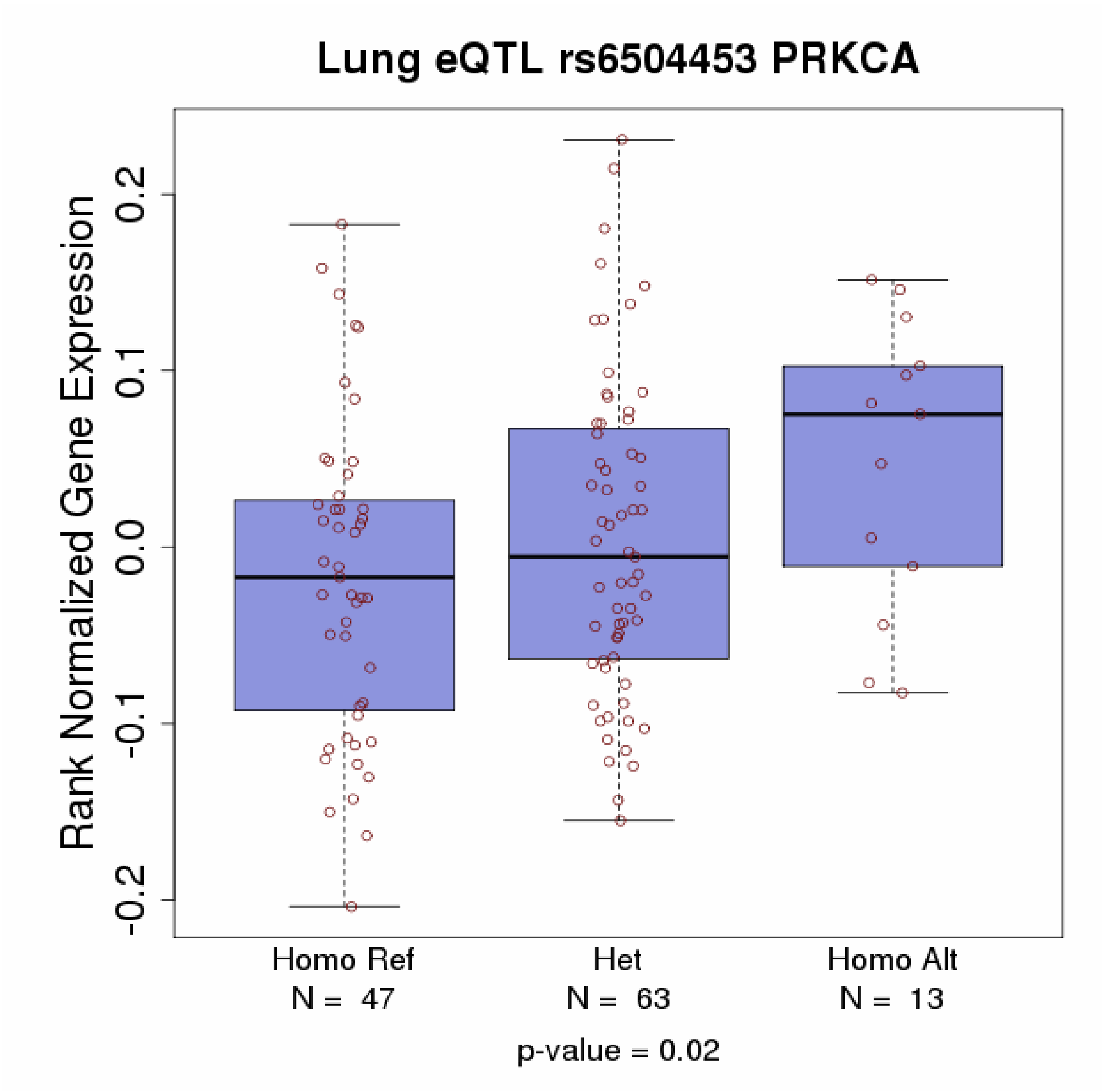

Figure S3. Association between T allele at rs6504453 in *PRKCA* and gene expression in lung tissue (eQTL browser GTEx, Gibson et al. 2015).
